# Kinetic effects regularize the mass-flux singularity at the contact line of a thin evaporating drop

**DOI:** 10.1007/s10665-016-9892-4

**Published:** 2017-01-23

**Authors:** M. A. Saxton, D. Vella, J. P. Whiteley, J. M. Oliver

**Affiliations:** 1 Department of Applied Mathematics and Theoretical Physics, University of Cambridge, Wilberforce Road, Cambridge, CB3 0WA UK; 2 Mathematical Institute, University of Oxford, Andrew Wiles Building, Radcliffe Observatory Quarter, Woodstock Road, Oxford, OX2 6GG UK; 3 Department of Computer Science, University of Oxford, Parks Road, Oxford, OX1 3QD UK

**Keywords:** Contact line, Evaporation, Kinetic effects, Mixed-boundary-value problems

## Abstract

We consider the transport of vapour caused by the evaporation of a thin, axisymmetric, partially wetting drop into an inert gas. We take kinetic effects into account through a linear constitutive law that states that the mass flux through the drop surface is proportional to the difference between the vapour concentration in equilibrium and that at the interface. Provided that the vapour concentration is finite, our model leads to a finite mass flux in contrast to the contact-line singularity in the mass flux that is observed in more standard models that neglect kinetic effects. We perform a local analysis near the contact line to investigate the way in which kinetic effects regularize the mass-flux singularity at the contact line. An explicit expression is derived for the mass flux through the free surface of the drop. A matched-asymptotic analysis is used to further investigate the regularization of the mass-flux singularity in the physically relevant regime in which the kinetic timescale is much smaller than the diffusive one. We find that the effect of kinetics is limited to an inner region near the contact line, in which kinetic effects enter at leading order and regularize the mass-flux singularity. The inner problem is solved explicitly using the Wiener–Hopf method and a uniformly valid composite expansion is derived for the mass flux in this asymptotic limit.

## Introduction

The evaporation of a liquid drop on a solid substrate has many important biomedical, geophysical, and industrial applications. Such applications include DNA mapping and gene-expression analysis, the water cycle, and the manufacture of semiconductor and micro-fluidic devices (see, for example, [1–7] and references therein). Modelling mass transfer from a partially wetting liquid drop is complicated because one must consider the transport of mass, momentum, and energy within and between three phases: the solid substrate, the liquid, and the surrounding atmosphere (assumed here to be a mixture of the liquid vapour and an inert gas). A key ingredient of any such model is an expression for the mass flux across the liquid–gas interface.

A commonly used model of a drop evaporating into an inert gas is the ‘lens’ model [2, 5, 8–12]. The lens model is based on the assumptions that the drop is axisymmetric, the vapour concentration field is stationary, and the vapour immediately above the liquid–gas interface is at thermodynamic equilibrium, with the equilibrium vapour concentration being constant. These assumptions imply that evaporation is limited by the diffusion of vapour away from the interface. Notably, however, the lens model is thought not to apply to water [2, 11].

The ‘lens’ model is so-called because the mixed-boundary-value problem for the vapour concentration is mathematically equivalent to that of finding the electric potential around a lens-shaped conductor [10, 13]. Furthermore, if the drop is thin, this problem reduces to one equivalent to that of finding the electric potential around a disc charged to a uniform potential. The analytical solution of this electrostatic problem [14], translated to the evaporation problem, shows that the mass flux $E^*$ per unit area per unit time has the form

1 $$\begin{aligned} E^* \propto \frac{1}{(R^2 - r^{*2})^{1/2}}, \end{aligned}$$

where *R* is the radius of the circular contact set and $r^*$ is the distance from the axis of symmetry of the thin drop. The expression (1) for the mass flux has an inverse-square-root singularity at the contact line. Since this singularity is integrable, the total mass flux out of the drop is not singular, and physically reasonable predictions for the evolution of the drop volume are obtained even without regularization of the mass-flux singularity [10, 12]. However, the need to supply a diverging mass flux means that there is a singularity in the depth-averaged radial velocity of the liquid flow within the drop [10, 12]. Such a divergent velocity is clearly unphysical. In reality the mass flux at the contact line must be finite. Relaxing the assumption that the vapour concentration is stationary affects only the coefficient of the singularity. Instead, the assumption that the vapour immediately above the liquid–gas interface is at equilibrium must be invalid in the vicinity of the contact line.

If the gas phase surrounding the drop instead consists of its vapour only (and no inert gas), an alternative boundary condition to apply on the liquid–gas interface is the Hertz–Knudsen relation, derived from the kinetic theory of gases [15]. The Hertz–Knudsen relation states that the mass flux across the drop surface per unit area per unit time is proportional to the difference between the equilibrium vapour density and the density of the vapour immediately above the drop. Formulated in terms of the vapour concentration (rather than the vapour density), on the free surface of the drop, we have

2 $$\begin{aligned} E^* = Mv_\mathrm{{k}}(c_\mathrm{{e}}^* - c^*), \end{aligned}$$

where *M* is the molar mass of the liquid vapour, $v_\mathrm{{k}}$ is a typical kinetic velocity (which we define later in the paper), $c_\mathrm{{e}}^*$ is the equilibrium vapour concentration, and $c^*$ is the vapour concentration at the interface. It is immediately apparent from the expression (2) that, provided the vapour concentration $c^*$ is finite, the mass flux is non-singular. The Hertz–Knudsen relation or the modified versions formulated in terms of vapour pressure, density, or temperature, have previously been used to model the evaporation of thin films [16], vapour bubbles in microchannels [17], and droplet evaporation on a precursor film [18]. While the assumptions required to derive the Hertz–Knudsen relation are not strictly satisfied when an inert gas is present, there is some experimental evidence that the Hertz–Knudsen relation is valid in such situations [19]. A possible explanation for this is that immediately above the drop, the gas phase is almost entirely vapour. It may therefore be reasonable to use the Hertz–Knudsen relation to model evaporation into an inert gas [20, 21].

To close a model based upon the Hertz–Knudsen relation (2), it is necessary to prescribe a constitutive law for the equilibrium vapour concentration $c_\mathrm{{e}}^*$ (of course, such a constitutive law is also necessary if one makes the equilibrium assumption that $c^* = c_\mathrm{{e}}^*$ on the liquid–gas interface). The simplest choice of constitutive law is to assume that the equilibrium vapour concentration is constant (as in the lens model). For a constant equilibrium vapour concentration, a kinetics-based model has the major advantage that, to leading order in the thin-film limit, the vapour transport problem depends on the liquid flow solely through the geometry of the contact set (and not through the drop thickness). This means that the vapour transport problem may be solved independently of the liquid problem. In this study, we shall exploit the simplicity of a kinetics-based model with a constant equilibrium vapour concentration to perform a mathematical analysis of the model and investigate the way in which kinetic effects regularize the mass-flux singularity.

Another possible constitutive law for the equilibrium vapour concentration is Kelvin’s equation; this takes into account the variation in vapour pressure due to the curvature of the liquid–gas interface [22]. This approach has been used to model the evaporation of liquid drops in the presence of an ultra-thin precursor film that wets the substrate ahead of the drop [8, 23]. In the bulk of the drop (away from the contact line), the dominant term in a linearized version of Kelvin’s equation is independent of the drop thickness. As a result, in an outer region away from the contact line, a constant vapour concentration is prescribed on the liquid–gas interface and the mass flux appears to have a singularity at the contact line [23]. This singularity is in fact regularized in an inner region in the vicinity of the contact line, in which the other terms in Kelvin’s equation become important [24]. In problems with a moving contact line, this evaporation model has the significant advantage that it also regularizes the stress singularity at the contact line [25, 26]. Another advantage is the compatibility of the model with a precursor film; there is experimental evidence that such films exist in at least some parameter regimes [27, 28]. We shall neglect the Kelvin effect in this paper, and establish *a posteriori* the regimes in which it is appropriate to do so (see Appendix 6).

In this paper, we adopt a linear, kinetics-based constitutive law for the mass flux across the liquid–gas interface, inspired by the Hertz–Knudsen relation (2); we assume that the equilibrium vapour concentration is constant. We will have two main goals. The first is to investigate the way in which kinetic effects regularize the mass-flux singularity at the contact line. The second is to derive an explicit expression for the evaporation rate. In Sect. 2, we formulate and non-dimensionalize the mixed-boundary-value problem for the vapour concentration. In Sect. 3, we perform a local analysis of both the lens evaporation model and the kinetics-based model to investigate the regularization of the mass-flux singularity at the contact line. In Sect. 4, we solve the mixed-boundary-value problem formulated in Sect. 2 to obtain an explicit expression for the evaporation rate. In Sect. 5, we perform an asymptotic analysis in the physically relevant limit in which the timescale of vapour diffusion is much longer than the timescale of kinetic effects to gain further insight into how kinetic effects regularize the mass-flux singularity. We find that there is an outer region away from the contact line where the equilibrium assumption (which leads to the mass-flux singularity) is recovered from our constitutive law and an inner region near the contact line where kinetic effects regularize the mass-flux singularity. The inner problem is solved explicitly using the Wiener–Hopf method, allowing us to derive a uniformly valid composite expansion for the mass flux in this asymptotic limit. In Sect. 6, we summarize our results and outline some possible directions for future work.

## Formulation

**Fig. 1:**
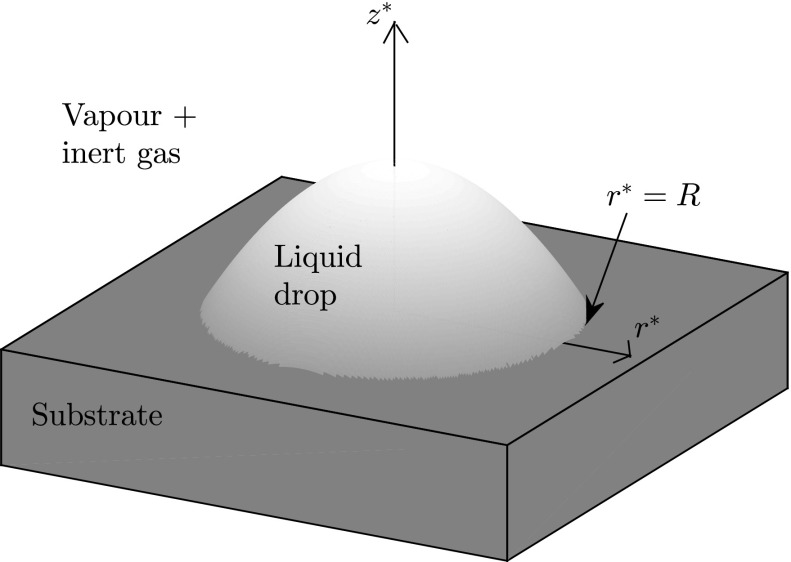
Definition sketch. Cylindrical polar coordinates $(r^*,z^*)$ measure the radial distance from the axis of symmetry of the drop and the normal distance from the substrate, respectively. The location of the contact line is $(r^*,z^*) = (R,0)$.

We consider a three-dimensional, axisymmetric drop on a rigid, flat, impermeable substrate. We introduce cylindrical polar coordinates $(r^*,z^*)$ measuring the radial distance from the axis of symmetry of the drop and the normal distance from the substrate, respectively (here and hereafter, starred variables denote dimensional quantities). The contact set of the drop is $0 \le r^* < R$, so that $(r^*,z^*) = (R,0)$ is the location of the contact line (at which the drop thickness vanishes). A mixture of liquid vapour and an inert gas occupies the region above the drop and substrate. A definition sketch is shown in Fig. 1. We assume that the drop is thin: the slope everywhere is comparable to the microscopic contact angle, $\varPhi \ll 1$. Thus, the vertical extent of the drop is much smaller than the radius of the circular contact set of the drop; since the latter is the relevant lengthscale for the transport of liquid vapour, the gas phase occupies the region $z^* > 0$ to leading order in the limit of a thin drop.

We assume that the dynamics of the vapour may be reduced to a diffusion equation for the vapour concentration $c^*$, with constant diffusion coefficient *D*. We further assume that the timescale of vapour diffusion is much shorter than the timescale of the liquid flow (a common assumption in the literature [9, 10, 12]). Thus, transport of the vapour is governed to leading order in the thin-film limit by Laplace’s equation, with

3 $$\begin{aligned} \nabla ^2c^* = 0 \quad \hbox {for}~z^* > 0. \end{aligned}$$

We assume that the vapour concentration in the far field takes a constant value $c_{\infty }$, so that

4 $$\begin{aligned} c^* \rightarrow c_\infty \quad \hbox {as}~r^{*2} + z^{*2} \rightarrow \infty ,~z^* > 0. \end{aligned}$$

The inert gas is assumed to be insoluble in the liquid, so that the mass flux $E^*$ across the interface per unit area per unit time is entirely accounted for by the mass flux of liquid vapour. Since the substrate is impermeable, we have a condition of no flux of vapour through the substrate. After linearizing the boundary condition on the surface of the drop onto $z^* = 0$, we obtain, to leading order in the thin-film limit, the boundary conditions

5 $$\begin{aligned}&-DM\frac{\partial {c^*}}{\partial {z^*}} = E^* \quad \hbox {on}~z^* = 0,~0 \le r^* < R, \end{aligned}$$

6 $$\begin{aligned}&\frac{\partial {c^*}}{\partial {z^*}} = 0 \quad \hbox {on}~z^* = 0,~r^* > R, \end{aligned}$$

where *M* is the molar mass of the liquid vapour.

We assume that the mass flux out of the drop is governed by a linear constitutive law, given by

7 $$\begin{aligned} E^* = Mv_\mathrm{{k}}(c_\mathrm{{e}} - c^*), \end{aligned}$$

where the equilibrium vapour concentration $c_\mathrm{{e}}$ is a constant. The constitutive law (7) is inspired by the Hertz–Knudsen relation [15]. As discussed in Sect. 1, the Hertz–Knudsen relation is strictly only valid when the gas phase consists of pure vapour. However, there is experimental evidence that it may be valid for a vapour–inert gas mixture [19], and it has previously been used to model such situations [20, 21]. The constant $v_\mathrm{{k}}$ is a typical kinetic velocity, given by

8 $$\begin{aligned} v_\mathrm{{k}} = \sigma _\mathrm{{e}}\left( \frac{R_\mathrm{{u}}T_\mathrm{in}}{2\pi M}\right) ^{1/2}, \end{aligned}$$

where $R_\mathrm{{u}}$ is the universal gas constant and $T_\mathrm{in}$ is the interfacial temperature. The (dimensionless) evaporation coefficient $\sigma _\mathrm{{e}}$ is the fraction of the maximum possible evaporating flow rate that actually occurs [15]. One disadvantage of the constitutive law (7) is that the evaporation coefficient $\sigma _\mathrm{{e}}$ is difficult to estimate; although a value of unity has been reported for many standard liquids, smaller values (anywhere between about $10^{-4}$ and 1) have been reported in other cases.

A quantity of interest is the surface-integrated flux out of the drop $Q^*$, given by

9 $$\begin{aligned} Q^* = 2\pi \int _0^R r^*E^*(r^*)\,\mathrm {d}r^*. \end{aligned}$$

The quantity $Q^*$ is needed to determine the evolution of the volume of the drop and thus the extinction time (at which the drop volume vanishes), even in models that do not consider the detailed hydrodynamics of motion [29, 30].

We see that if the contact line is pinned (so that the contact-set radius *R* is constant) the model (3)–(7) is independent of time—i.e. the problem is steady. If instead the contact line is allowed to move (so that *R* depends on time), then the problem is quasi-steady; the time dependence would become important if the expression that we ultimately derive for the mass flux were to be used as an input for a model for the evolution of the liquid drop. We shall use the contact-set radius *R* as a typical lengthscale on which to non-dimensionalize, suppressing the dependence of *R* on time in the case that the contact line is allowed to move. Thus, the expression that we shall ultimately derive for the evaporation rate will be valid for drops with either pinned or moving contact lines.

We non-dimensionalize (3)–(7) by scaling $r^* = Rr$, $z^* = Rz$, $c^* = c_\infty + (c_\mathrm{{e}} - c_\infty )c$, and $E^* = DM(c_\mathrm{{e}}-c_\infty )E/R$. We obtain thereby the following mixed-boundary-value problem for the dimensionless vapour concentration *c*(*r*, *z*):

10 $$\begin{aligned}&\nabla ^2 c = 0 \quad \hbox {for}~z > 0, \end{aligned}$$

11 $$\begin{aligned}&c \rightarrow 0 \quad \hbox {as}~r^2 + z^2 \rightarrow \infty ,~z > 0, \end{aligned}$$

12 $$\begin{aligned}&-\frac{\partial {c}}{\partial {z}} = \mathrm {Pe_k}(1-c) \quad \hbox {on}~z = 0,~0 \le r < 1, \end{aligned}$$

13 $$\begin{aligned}&\frac{\partial {c}}{\partial {z}} = 0 \quad \hbox {on}~z = 0,~r > 1, \end{aligned}$$

where non-dimensionalization has introduced a dimensionless parameter, namely the kinetic Péclet number,

14 $$\begin{aligned} \mathrm {Pe_k}= \frac{Rv_\mathrm{{k}}}{D}. \end{aligned}$$

The kinetic Péclet number is the ratio of the timescales of diffusive and kinetic effects (over the radius of the circular contact set of the drop: $R^2/D$ and $R/v_\mathrm{{k}}$, respectively) and is the only parameter remaining in the problem following non-dimensionalization. We note the physical significance of two extreme cases: $\mathrm {Pe_k}= 0$ corresponds to the case of no mass transfer, while $\mathrm {Pe_k}= \infty$ corresponds to the case in which the vapour immediately above the free surface is at thermodynamic equilibrium, so that $c = 1$ on $z=0,~0\le r<1$. Since this is the limit used in the lens model, we expect to obtain a diverging mass flux at the contact line as $\mathrm {Pe_k}\rightarrow \infty$ (as will be discussed in Sect. 3.1). In Table 1, we give typical values of the relevant physical parameters for various liquids and various drop radii. We see that the kinetic Péclet number may take a wide range of values, but that it is at least moderately large for all but very small drops.

**Table 1:** Values of the physical parameters used in the model for hexane, isopropanol, and HFE-7100 at 25 $^{\circ }$C and 1 atm [11, 21, 31, 32]

	Hexane	Isopropanol	HFE-7100
*D* (cm$^2$, s$^{-1}$)	0.03	0.096	0.061
*M* (g mol$^{-1}$)	86.2	60.1	250
$c_\mathrm{{e}}$ (mol m$^{-3}$)	0.02	2.2	10.9
$v_\mathrm{{k}}$ (m s$^{-1}$)	67.6	81.0	28.1
$\mathrm {Pe_k},R = 1$ mm (–)	$2.2\times 10^4$	$8.4\times 10^3$	$4.6\times 10^3$
$\mathrm {Pe_k},R = 10\,\upmu$m (–)	220	84	46

The equilibrium vapour concentration $c_\mathrm{{e}}$ is evaluated using the saturation vapour pressure. In calculating the typical kinetic velocity $v_\mathrm{{k}}$ from (8), we assume that the evaporation coefficient $\sigma _\mathrm{{e}} = 1$ and that the interfacial temperature $T_\mathrm{in}$ is constant at 25 $^{\circ }$C. We assume that $c_\infty = 0$ for each of the liquids in the table. The kinetic Péclet number $\mathrm {Pe_k}= Rv_\mathrm{{k}}/D$ is given for (thin) drops with contact-set radii $R=1$ mm and $R = 10\,\upmu$m

The key quantity of interest, the dimensionless evaporation rate *E*(*r*), is given by

15 $$\begin{aligned} E(r) = -\left. \frac{\partial {c}}{\partial {z}}\right| _{z = 0} = \mathrm {Pe_k}[1-c(r,0)] \quad \hbox {for}~0 \le r < 1. \end{aligned}$$

A related quantity of interest, and a useful proxy, is the evaporation rate at the contact line, $E(1^-)$; the liquid motion has a strong dependence upon the size of this quantity [33]. We note that with $\mathrm {Pe_k}= \infty$, $E(1^-)$ is not defined. Non-dimensionalization implies that $Q^* = DM(c_\mathrm{{e}}-c_\infty )RQ$, where the total (dimensionless) flux out of the drop *Q* is given by

16 $$\begin{aligned} Q = 2\pi \int _0^1 rE(r)\,\mathrm {d}r. \end{aligned}$$

We emphasize that the three quantities *E*(*r*), $E(1^-)$, and *Q* are all functions of the kinetic Péclet number $\mathrm {Pe_k}$. They therefore depend on the contact-set radius *R* (but not, in the thin-film limit, on the drop thickness).

## Local analysis near the contact line

In this section, we perform a local analysis near the contact line of both the lens model and the kinetics-based model (considering the former puts the latter into context). This will demonstrate explicitly that the lens model has a mass-flux singularity at the contact line, while the kinetics-based model does not. Comparing the local expansions for the two models should also give us some insight into the way in which the kinetics-based model regularizes the mass-flux singularity.

### Lens model

For the lens model, the boundary condition (12) is replaced by

17 $$\begin{aligned} c = 1 \quad \hbox {on}~\quad z = 0,~0 \le r < 1. \end{aligned}$$

As noted earlier, this may be viewed as a special case of (12) with $\mathrm {Pe_k}= \infty$. Recall that the lens model (10), (11), (13), and (17) is mathematically equivalent to the problem of finding the electric potential around a disc charged to a uniform potential [14]. Assuming continuity of *c* at $r = 1$, this electrostatic problem has an exact solution [34, 35], given by

18 $$\begin{aligned} c(r,z) = \frac{2}{\pi }\sin ^{-1}\left( \frac{2}{\left( (r-1)^2+z^2\right) ^{1/2} + \left( (r+1)^2 + z^2\right) ^{1/2}}\right) . \end{aligned}$$

We deduce from (18) that the evaporation rate is given by

19 $$\begin{aligned} E(r) = \frac{2}{\pi (1-r^2)^{1/2}} \quad \hbox {for}~0 \le r < 1. \end{aligned}$$

We note from (19) that the total flux, $Q = 4$, is finite.

From the exact solution (18), we deduce that the local expansion of the solution near the contact line is given by

20 $$\begin{aligned} c(r,z) \sim 1 - \frac{2^{3/2}}{\pi }\rho ^{1/2}\cos \left( \frac{\theta }{2}\right) , \end{aligned}$$

as $\rho \rightarrow 0^+,~0 \le \theta < \pi$, where $(\rho ,\theta \,)$ are local polar coordinates defined by $r = 1 + \rho \cos \theta$, $z = \rho \sin \theta$. The corresponding evaporation rate near the contact line has the local expansion

21 $$\begin{aligned} E(r) \sim \frac{2^{1/2}}{\pi (1-r)^{1/2}} \quad \hbox {as}~r \rightarrow 1^-. \end{aligned}$$

Thus, we see clearly that there is an inverse-square-root singularity in the evaporation rate at the contact line, $r = 1$. In Appendix 1, we show how this singularity leads to a singularity in the depth-averaged radial velocity of the liquid drop, which is unphysical.

### Kinetics-based model

We now return to the mixed-boundary-value problem (10)–(13) for finite $\mathrm {Pe_k}$. We assume that *c* is continuous at the contact line and takes the value $c_\mathrm{{L}}(\mathrm {Pe_k})$ there, with $c_\mathrm{{L}}(\mathrm {Pe_k})$ not equal to 0 or 1. Under these assumptions, a local analysis near the contact line implies that

22 $$\begin{aligned} c(r,z) \sim c_\mathrm{{L}}(\mathrm {Pe_k}) + \frac{\mathrm {Pe_k}[1-c_\mathrm{{L}}(\mathrm {Pe_k})]}{\pi }\rho [(\cos \theta )(\log \rho ) - \theta \sin \theta ], \end{aligned}$$

as $\rho \rightarrow 0^+$ for $0 \le \theta \le \pi$, where $c_\mathrm{{L}}(\mathrm {Pe_k})$ is a degree of freedom. We then use (15) to find that the local expansion for the evaporation rate *E*(*r*) near the contact line is given by

23 $$\begin{aligned} E(r) \sim \mathrm {Pe_k}[1-c_\mathrm{{L}}(\mathrm {Pe_k})]\left[ 1 + \frac{\mathrm {Pe_k}}{\pi }(1-r)\log (1-r)\right] \quad \hbox {as}~r \rightarrow 1^-. \end{aligned}$$

In particular, this implies that the evaporation rate at the contact line $E(1^-)$ is given by

24 $$\begin{aligned} E(1^-) = \mathrm {Pe_k}[1-c_\mathrm{{L}}(\mathrm {Pe_k})]. \end{aligned}$$

Thus, the evaporation rate at the contact line (and everywhere else) is finite. In Appendix 1, we show that the depth-averaged radial velocity of the liquid drop is also finite.

We recall that the lens model is a special case of the kinetics-based model with $\mathrm {Pe_k}=\infty$. Thus, for the local expansions (20) and (22) to be in agreement, it must be the case that

25 $$\begin{aligned} c_\mathrm{{L}}(\mathrm {Pe_k}) \rightarrow 1 \quad \hbox {as}~\mathrm {Pe_k}\rightarrow \infty , \end{aligned}$$

but with $c_\mathrm{{L}} < 1$ for finite $\mathrm {Pe_k}$. Hence, we will be interested in determining the degree of freedom $c_\mathrm{{L}}(\mathrm {Pe_k})$ by solving the mixed-boundary-value problem (10)–(13).

## Explicit expression for the evaporation rate

We shall now solve the mixed-boundary-value problem (10)–(13). An important aim of this calculation is to determine the degree of freedom $c_\mathrm{{L}}(\mathrm {Pe_k})$, appearing in (22), which will put the results of Sect. 3 in context. We will also obtain an explicit expression for the evaporation rate; this expression would be a key ingredient in investigations of the evolution of the drop.

### Solution of the mixed-boundary-value problem

We note that the mixed-boundary-value problem (10)–(13) is mathematically equivalent to that of finding the temperature around a partially thermally insulated disc whose exterior is completely insulated; this problem was solved by Gladwell et al. [36] using Hankel, Fourier cosine, and Abel transforms, as well as properties of Legendre polynomials. The solution is given by

26 $$\begin{aligned} c(r,z) = \int _0^\infty \int _0^1 f(x)\cos (kx)J_0(kr)\mathrm{{e}}^{-kz}\,\mathrm {d}x\,\mathrm {d}k, \end{aligned}$$

where $J_0(kr)$ is the Bessel function of first kind of order zero, and the function *f*(*x*) satisfies the Abel integral equation given by

27 $$\begin{aligned} -\frac{1}{\mathrm {Pe_k}}\frac{1}{r}\frac{\mathrm {d}{}}{\mathrm {d}{r}}\int _r^1 \frac{xf(x)}{(x^2-r^2)^{1/2}}\,\mathrm {d}x + \int _0^r \frac{f(x)}{(r^2-x^2)^{1/2}}\,\mathrm {d}x = 1 \quad \hbox {for}~0< r < 1. \end{aligned}$$

By writing $f(x) = \sum _{n=0}^\infty a_n\sin [(2n+1)\cos ^{-1}(x)]$ and expanding (27) in Legendre polynomials [36], we obtain

28 $$\begin{aligned} c(r,z) = \sum _{n=0}^\infty a_n(\mathrm {Pe_k})\int _0^\infty \int _0^1 \sin [(2n+1)\cos ^{-1}x]\cos (kx)\mathrm{{e}}^{-kz}J_0(kr)\,\mathrm {d}x\,\mathrm {d}k, \end{aligned}$$

where the coefficients $a_n(\mathrm {Pe_k})$ satisfy a system of infinitely many linear algebraic equations, given by

29 $$\begin{aligned} \frac{(2n+1)\pi }{2\mathrm {Pe_k}}a_n(\mathrm {Pe_k}) + \sum _{m=0}^\infty b_{mn}a_m(\mathrm {Pe_k}) = \delta _{0n} \quad \hbox {for}~n = 0,1,2,\ldots , \end{aligned}$$

where

30 $$\begin{aligned} b_{mn} = \frac{1}{2}\left( \frac{1}{2m+2n+1}+\frac{1}{2m-2n+1}-\frac{1}{2m+2n+3}-\frac{1}{2m-2n-1}\right) , \end{aligned}$$

and $\delta _{0n}$ is the Kronecker delta.

Using (15) we deduce that, for $0 \le r < 1$, the evaporation rate is given by

31 $$\begin{aligned} E(r) = \sum _{n=0}^\infty a_n(\mathrm {Pe_k})\int _0^\infty \int _0^1 \sin [(2n+1)\cos ^{-1}(x)]k\cos (kx)J_0(kr)\,\mathrm {d}x\,\mathrm {d}k. \end{aligned}$$

We integrate by parts once with respect to *x* and then change the order of integration. The resulting integral with respect to *k* may be evaluated explicitly, yielding

32 $$\begin{aligned} E(r) = \sum _{n=0}^\infty (2n+1)a_n(\mathrm {Pe_k}) \int _r^1 \frac{\cos [(2n+1)\cos ^{-1}(x)]}{(x^2-r^2)^{1/2}(1-x^2)^{1/2}}\,\mathrm {d}x, \end{aligned}$$

for $0 \le r < 1$. From this expression it is not clear, without further analysis, how *E* behaves as the contact line is approached, i.e. as $r \rightarrow 1^-$.

In Appendix 2, we analyse (32) as $r\rightarrow 1^-$ to find that the evaporation rate at the contact line $E(1^-)$ is given by

33 $$\begin{aligned} E(1^-) = \frac{\pi }{2}\sum _{n=0}^\infty (2n+1)a_n(\mathrm {Pe_k}). \end{aligned}$$

By comparing the expression (33) for the evaporation rate at the contact line to the earlier expression (24) for the same quantity in terms of the concentration $c_\mathrm{{L}}(\mathrm {Pe_k})$ at the contact line, we deduce that

34 $$\begin{aligned} c_\mathrm{{L}}(\mathrm {Pe_k}) = 1 - \frac{E(1^-)}{\mathrm {Pe_k}} = 1 - \frac{\pi }{2\mathrm {Pe_k}}\sum _{n=0}^\infty (2n+1)a_n(\mathrm {Pe_k}). \end{aligned}$$

In practical applications, we may be interested in the total flux out of the drop, *Q*, given by

35 $$\begin{aligned} Q = 2\pi \sum _{n=0}^\infty (2n+1)a_n(\mathrm {Pe_k}) \int _0^1\int _r^1 \frac{r\cos [(2n+1)\cos ^{-1}(x)]}{(x^2-r^2)^{1/2}(1-x^2)^{1/2}}\,\mathrm {d}x\,\mathrm {d}r. \end{aligned}$$

We switch the order of integration since the integral with respect to *r* can be evaluated analytically. We then evaluate the remaining integral via the substitution $x = \cos (\theta )$ to obtain

36 $$\begin{aligned} Q = \frac{\pi ^2a_0(\mathrm {Pe_k})}{2}, \end{aligned}$$

which is finite (as is also the case for infinite kinetic Péclet number).

### Computing the evaporation rate

We have now deduced expressions for the evaporation rate *E*(*r*) for $0 \le r < 1$, the concentration $c_\mathrm{{L}}(\mathrm {Pe_k})$ at the contact line, the evaporation rate at the contact line $E(1^-)$, and the total flux out of the drop *Q* in terms of a set of coefficients $a_n(\mathrm {Pe_k})$ that satisfy a system of infinitely many linear algebraic equations (29). We shall now describe how to solve numerically this algebraic system and thus how to compute the evaporation rate in practice.

**Fig. 2:**
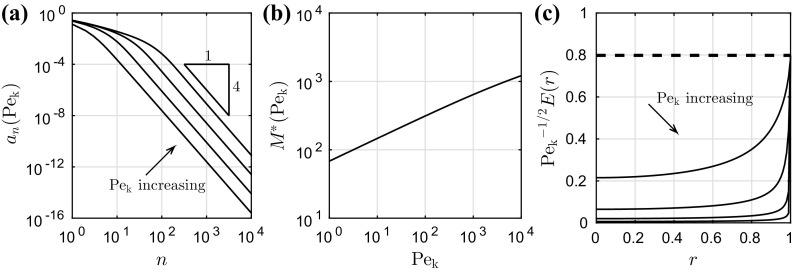
**a** The coefficients $a_n(\mathrm {Pe_k})$ that solve the algebraic system (29) truncated at $n = 10^4$, as a function of *n* for $\mathrm {Pe_k}= 10^1,10^2,10^3,10^4$. **b** The value $n = M^*(\mathrm {Pe_k})$ at which the algebraic system (29) should be truncated so that the truncation error (37) is below $10^{-4}$. **c** Scaled evaporation rate $\mathrm {Pe_k}^{-1/2}E(r)$ as a function of *r* for $\mathrm {Pe_k}= 10^1,10^2,10^3,10^4$. The dashed line shows the apparent large-$\mathrm {Pe_k}$ asymptote (38) for the evaporation rate at the contact line (details in text)

Previous work has shown that the system is regular [37] (in the sense that $a_{n+1} \ll a_n$ as $n\rightarrow \infty$) and may therefore be solved by truncation. In Fig. 2a, we plot $a_n(\mathrm {Pe_k})$ as a function of *n* for several values of $\mathrm {Pe_k}$. We observe that $a_n = \mathrm {O}(n^{-4})$ as $n\rightarrow \infty$; this rapid decay confirms that truncating the system (at a suitably large value of *n*) is appropriate.

It remains to determine a suitable value of *n* at which to truncate the system (29). We define the truncation error $T_M(\mathrm {Pe_k})$ in the evaporation rate at the contact line by

37 $$\begin{aligned} T_M(\mathrm {Pe_k}) = \frac{E_{2M}(\mathrm {Pe_k}) - E_M(\mathrm {Pe_k})}{E_{2M}(\mathrm {Pe_k})}, \quad E_M(\mathrm {Pe_k}) = \sum _{n=0}^M (2n+1)a_n(\mathrm {Pe_k}), \end{aligned}$$

where the coefficients $a_n$ satisfy the system (29) truncated at $n = M$. We define $M^*(\mathrm {Pe_k})$ to be the smallest value of *M* for which $T_M(\mathrm {Pe_k}) \le 10^{-4}$. We calculate $M^*$ for a range of values of $\mathrm {Pe_k}$ to create a lookup table, and then the value of $M^*$ for general $\mathrm {Pe_k}$ is determined by spline interpolation (rounding up to the nearest integer). We plot $M^*$ as a function of $\mathrm {Pe_k}$ in Fig. 2b. Thus, to compute the coefficients $a_n(\mathrm {Pe_k})$ in practice, we first use a lookup table and spline interpolation to determine a suitable value $n = M^*(\mathrm {Pe_k})$ at which to truncate the system (29). The resulting finite linear algebraic system is then solved using Matlab’s backslash command (since the system is symmetric positive definite, this uses Cholesky factorization).

Once the coefficients $a_n(\mathrm {Pe_k})$ have been determined numerically, the evaporation rate *E*(*r*) is approximated by (32) with the sum truncated at $n = M^*(\mathrm {Pe_k})$. The integral in (32) is evaluated numerically using the integral command in Matlab. We check convergence in the usual way by reducing the error tolerances. We plot a scaled evaporation rate $\mathrm {Pe_k}^{-1/2}E(r)$ as a function of *r* for several values of $\mathrm {Pe_k}$ in Fig. 2c. We see that the evaporation rate is everywhere finite for the values of $\mathrm {Pe_k}$ plotted (which we note from Table 1 covers physically realistic values).

We note from Fig. 2c that for large values of $\mathrm {Pe_k}$ there appears to be a boundary layer near to the contact line in which the evaporation rate is much larger. We also observe from Fig. 2c that there appears to be a large-$\mathrm {Pe_k}$ asymptote for the evaporation rate at the contact line of the form

38 $$\begin{aligned} E(1^-) \sim \alpha \mathrm {Pe_k}^{1/2} \quad \hbox {as}~\mathrm {Pe_k}\rightarrow \infty , \end{aligned}$$

for some constant $\alpha \approx 0.798$ (with this asymptote presented as the dashed line in Fig. 2c). We deduce from (34) that $c_\mathrm{{L}}(\mathrm {Pe_k}) < 1$ for finite $\mathrm {Pe_k}$ and that $c_\mathrm{{L}} \rightarrow 1^-$ as $\mathrm {Pe_k}\rightarrow \infty$, in agreement with our local analysis. Together with the fact that $\mathrm {Pe_k}$ is typically large in practice (see Table 1), this motivates us to undertake an asymptotic analysis of the limit $\mathrm {Pe_k}\rightarrow \infty$. It is not obvious how to find the coefficients $a_n(\mathrm {Pe_k})$ as $\mathrm {Pe_k}\rightarrow \infty$ in the algebraic system (29), nor is it obvious how to analyse the integral equation (27) as $\mathrm {Pe_k}\rightarrow \infty$, so we instead proceed by analysing the mixed-boundary-value problem (10)–(13) rather than the exact solution (32).

## Asymptotic analysis in the limit of large kinetic Péclet number

In this section, we perform a matched-asymptotic analysis of the limit $\mathrm {Pe_k}\rightarrow \infty$ to gain further insight into the way in which kinetic effects regularize the mass-flux singularity at the contact line. This is a singular perturbation problem; the asymptotic structure consists of an outer region in which $|1 - r|,~z = \mathrm {O}(1)$ as $\mathrm {Pe_k}\rightarrow \infty$, and an inner region near the contact line in which there is a full balance of terms in the boundary condition (12) on the free surface of the drop. We see that this happens when $z = \mathrm {O}(\mathrm {Pe_k}^{-1})$ and that to keep a full balance of terms in Laplace’s equation (10) we require $|1 - r| = \mathrm {O}(\mathrm {Pe_k}^{-1})$ as $\mathrm {Pe_k}\rightarrow \infty$.

### Outer region

We expand $c\sim c_0$ as $\mathrm {Pe_k}\rightarrow \infty$. We find that the leading-order vapour concentration $c_0(r,z)$ satisfies (10), (11), and (13), but the boundary condition (12) is replaced by

39 $$\begin{aligned} c_0 = 1 \quad \hbox {on}~z = 0,~0 \le r < 1. \end{aligned}$$

The leading-order vapour concentration therefore satisfies the mixed-boundary-value problem considered in Sect. 3.1 and we deduce that as $\mathrm {Pe_k}\rightarrow \infty$ with $(1-r)=\mathrm {O}(1)$,

40 $$\begin{aligned} E(r) \sim -\left. \frac{\partial {c_0}}{\partial {z}}\right| _{z=0} = \frac{2}{\pi (1-r^2)^{1/2}} \quad \hbox {for}~0 \le r < 1. \end{aligned}$$

We see that this outer evaporation rate has an inverse-square-root singularity as $r\rightarrow 1^-$; we expect this singularity to be regularized in an inner region close to $r = 1$.

### Inner region

#### The leading-order-inner problem

In an inner region near the contact line, we set $r = 1 + \mathrm {Pe_k}^{-1}X$, $z = \mathrm {Pe_k}^{-1}Y$, and expand $c(r,z) \sim 1 - \mathrm {Pe_k}^{-1/2}C(X,Y)$ as $\mathrm {Pe_k}\rightarrow \infty$. To leading order, the vapour transport equation (10) and the mixed-boundary conditions (12) and (13) become

41 $$\begin{aligned}&\frac{\partial ^2 C}{\partial X^2} + \frac{\partial ^2 C}{\partial Y^2} = 0 \quad \hbox {for}~Y > 0, \end{aligned}$$

42 $$\begin{aligned}&\frac{\partial {C}}{\partial {Y}} = C \quad \hbox {on}~Y = 0,~X < 0, \end{aligned}$$

43 $$\begin{aligned}&\frac{\partial {C}}{\partial {Y}} = 0 \quad \hbox {on}~Y = 0,~X > 0. \end{aligned}$$

Finally, matching with the leading-order-outer solution (18) gives the conditions

44 $$\begin{aligned} C \sim {\left\{ \begin{array}{ll} \dfrac{2^{3/2}}{\pi }\rho ^{1/2}\cos \dfrac{\theta }{2} \quad &{}\hbox {for}~0 \le \theta < \pi ,\\ \dfrac{2^{1/2}}{\pi }\rho ^{-1/2} \quad &{}\hbox {for}~\theta = \pi , \end{array}\right. } \end{aligned}$$

as $\rho \rightarrow \infty$, where $(\rho ,\theta )$ are now plane polar coordinates related to (*X*, *Y*) by $X = \rho \cos \theta$, $Y = \rho \sin \theta$.

A local analysis of (41) subject to (42) and (43), assuming *C* to be continuous and non-zero at the contact line, implies that

45 $$\begin{aligned} C(X,Y) \sim C_O\left\{ 1-\frac{\rho [(\log \rho )(\cos \theta ) - \theta \sin \theta ]}{\pi }\right\} \quad \hbox {as}~\rho \rightarrow 0^+,\quad 0 \le \theta \le \pi . \end{aligned}$$

The value of the leading-order-inner solution at the contact line, $C_O := C(0,0)$, is a degree of freedom in this expansion and we note that it is related to the degree of freedom $c_\mathrm{{L}}(\mathrm {Pe_k})$ in the local expansion (23) of the full problem by

46 $$\begin{aligned} c_\mathrm{{L}}(\mathrm {Pe_k}) \sim 1 - C_O\mathrm {Pe_k}^{-1/2} \quad \hbox {as}~\mathrm {Pe_k}\rightarrow \infty . \end{aligned}$$

The expression (24) for the evaporation rate at the contact line in terms of $c_\mathrm{{L}}(\mathrm {Pe_k})$ then tells us that

47 $$\begin{aligned} E(1^-) \sim C_O\mathrm {Pe_k}^{1/2} \quad \hbox {as}~\mathrm {Pe_k}\rightarrow \infty . \end{aligned}$$

We shall solve the mixed-boundary-value problem (41)–(44) using the Wiener–Hopf method. The methodology employed is analogous to that used by Thompson [38] to solve a similar problem (known as the ‘dock problem’) consisting of (41) and (43), but with a sign change to the right-hand side of (42) and with different far-field behaviours.

#### Regularized inner problem

We begin by defining the functions

48 $$\begin{aligned} C_+(X) = {\left\{ \begin{array}{ll} 0~&{}\hbox {for}~X< 0, \\ C(X,0)~&{}\hbox {for}~X \ge 0, \end{array}\right. } \quad C_-(X) = {\left\{ \begin{array}{ll} C(X,0)~&{}\hbox {for}~X < 0,\\ 0~&{}\hbox {for}~X \ge 0, \end{array}\right. } \end{aligned}$$

with corresponding one-sided Fourier transforms $\overline{C}_{\pm }(k)$ given by

49 $$\begin{aligned} \overline{C}_+(k) = \int _0^\infty C_+(X)\mathrm{{e}}^{ikX}\,\mathrm {d}X, \quad \overline{C}_-(k) = \int _{-\infty }^0 C_-(X)\mathrm{{e}}^{ikX}\,\mathrm {d}X. \end{aligned}$$

We shall assume (and verify a posteriori) that *C*(*X*, 0) is infinitely differentiable on $(-\infty ,0)$ and $(0,\infty )$. Then, using the far-field behaviour (44) and the local expansion (45), the Abelian Theorem in Appendix 3 tells us that $\overline{C}_+(k)$ is holomorphic in $\mathfrak {I}(k) > 0$, with

50 $$\begin{aligned} \overline{C}_+(k) \sim \frac{iC_O}{k} \quad \hbox {as}~k\rightarrow \infty ,~\mathfrak {I}(k) > 0, \end{aligned}$$

and $\overline{C}_-(k)$ is holomorphic in $\mathfrak {I}(k) < 0$, with

51 $$\begin{aligned} \overline{C}_-(k) \sim -\frac{iC_O}{k} \quad \hbox {as}~k\rightarrow \infty ,~\mathfrak {I}(k) < 0. \end{aligned}$$

Moreover, a standard asymptotic analysis implies that the behaviour of $\overline{C}_\pm (k)$ as $k\rightarrow 0$ is dominated by the behaviour of *C*(*X*, 0) as $X\rightarrow \pm \infty$, with

52 $$\begin{aligned} \overline{C}_+(k)\sim & {} \displaystyle \int _0^\infty \dfrac{2^{3/2}}{\pi }X^{1/2}\mathrm{{e}}^{ikX}\,\mathrm {d}X = \dfrac{2^{1/2}\mathrm{{e}}^{3\pi i/4}}{\pi ^{1/2}k_+^{3/2}} \quad \hbox {as}~k\rightarrow 0,~\mathfrak {I}(k) > 0, \end{aligned}$$

53 $$\begin{aligned} \overline{C}_-(k)\sim & {} \displaystyle \int _{-\infty }^0 \dfrac{2^{1/2}}{\pi }(-X)^{-1/2}\mathrm{{e}}^{ikX}\,\mathrm {d}X = \dfrac{2^{1/2}\mathrm{{e}}^{-i\pi /4}}{\pi ^{1/2}k_-^{1/2}} \quad \hbox {as}~k\rightarrow 0,~\mathfrak {I}(k) < 0, \end{aligned}$$

where $k_+^{3/2}$ and $k_-^{1/2}$ are defined as follows:

54 $$\begin{aligned} k_+^{3/2}= & {} |k|^{3/2}\mathrm{{e}}^{3i\arg (k)/2},~\hbox {for}~-\frac{\pi }{2} \le \arg (k) < \frac{3\pi }{2}, \end{aligned}$$

55 $$\begin{aligned} k_-^{1/2}= & {} |k|^{1/2}\mathrm{{e}}^{i\arg (k)/2},~\hbox {for}~-\frac{3\pi }{2} \le \arg (k) < \frac{\pi }{2}. \end{aligned}$$

Here, $k_+^{3/2}$ has a branch cut along the negative imaginary axis, while $k_-^{1/2}$ has a branch cut along the positive imaginary axis. The choice of branch is such that both $k_+^{3/2}$ and $k_-^{1/2}$ are real and positive when *k* is real and positive, so that $\overline{C}_+(k)$ is real and positive on the positive imaginary axis and $\overline{C}_-(k)$ is real and positive on the negative imaginary axis.

The Abelian Theorem tells us that there is no value of *k* for which both $\overline{C}_+(k)$ and $\overline{C}_-(k)$ exist, so we are unable to apply the Wiener–Hopf method to the problem as it stands. Instead, we consider in the usual way [39, 40] the regularized problem for the function $C^{\varepsilon }(X,Y)$, given by

56 $$\begin{aligned}&\frac{\partial ^2 C^{\varepsilon }}{\partial X^2} + \frac{\partial ^2 C^{\varepsilon }}{\partial Y^2} = \varepsilon ^2C^{\varepsilon }\quad \hbox {for}~Y > 0, \end{aligned}$$

57 $$\begin{aligned}&\frac{\partial {C^{\varepsilon }}}{\partial {Y}} = C^{\varepsilon }\quad \hbox {on}~Y = 0,~X < 0, \end{aligned}$$

58 $$\begin{aligned}&\frac{\partial {C^{\varepsilon }}}{\partial {Y}} = 0 \quad \hbox {on}~Y = 0,~X > 0. \end{aligned}$$

We shall subsequently take the limit $\varepsilon \rightarrow 0^+$ to recover the leading-order-inner solution $C(X,Y) = \lim _{\varepsilon \rightarrow 0^+}C^{\varepsilon }(X,Y)$.

A local analysis of (56) subject to (57) and (58), assuming $C^\varepsilon$ to be continuous and non-zero at the contact line, implies that $C^{\varepsilon }(X,Y)$ has the same local expansion (45) at the origin as *C*(*X*, *Y*), but with $C_O$ replaced by $C^{\varepsilon }_O:=C^{\varepsilon }(0,0)$. A far-field analysis, admitting only exponentially decaying separable solutions, implies that we require

59 $$\begin{aligned} \frac{\partial {C^{\varepsilon }}}{\partial {X}} \sim {\left\{ \begin{array}{ll} \dfrac{A^{\varepsilon }\mathrm{{e}}^{-\varepsilon \rho }}{\rho ^{1/2}}\cos \dfrac{\theta }{2} \quad &{}\hbox {for}~0\le \theta < \pi ,\\ \dfrac{A^{\varepsilon }\mathrm{{e}}^{-\varepsilon \rho }}{2\rho ^{3/2}} \quad &{}\hbox {for}~\theta = \pi , \end{array}\right. } \end{aligned}$$

as $\rho \rightarrow \infty$, where in order to recover (44) in the limit $\varepsilon \rightarrow 0^+$, it is necessary for the constant $A^\varepsilon$ to satisfy the condition

60 $$\begin{aligned} \lim _{\varepsilon \rightarrow 0^+} A^{\varepsilon } = \frac{2^{1/2}}{\pi }. \end{aligned}$$

We now define $F^\varepsilon (X) = \partial C^{\varepsilon }/\partial X (X,0)$. Using (59), we deduce from the Abelian Theorem in Appendix 3 that $\overline{F}^\varepsilon _+(k)$ is holomorphic in $\mathfrak {I}(k) > -\varepsilon$, while $\overline{F}^\varepsilon _-(k)$ is holomorphic in $\mathfrak {I}(k) < \varepsilon$. Moreover, we have

61 $$\begin{aligned} \overline{F}^\varepsilon _+(k)= & {} -ik\overline{C}^{\varepsilon }_+(k) - C^{\varepsilon }_O\quad \hbox {for}~\mathfrak {I}(k) > 0, \end{aligned}$$

62 $$\begin{aligned} \overline{F}^\varepsilon _-(k)= & {} -ik\overline{C}^{\varepsilon }_-(k) + C^{\varepsilon }_O\quad \hbox {for}~\mathfrak {I}(k) < 0, \end{aligned}$$

where the functions $C^\varepsilon _\pm (X)$ and their Fourier transforms $\overline{C}^\varepsilon _\pm (k)$ are defined analogously to (48) and (49). By applying analytic continuation, we deduce that $\overline{C}^{\varepsilon }_+(k)$ is holomorphic in $\mathfrak {I}(k) > -\varepsilon$ except for a simple pole at $k = 0$ and $\overline{C}^{\varepsilon }_-(k)$ is holomorphic in $\mathfrak {I}(k) < \varepsilon$ except for a simple pole at $k = 0$. The presence of a simple pole at the origin in both $\overline{C}^{\varepsilon }_+(k)$ and $\overline{C}^{\varepsilon }_-(k)$ is consistent with the constants $a_\pm$ being non-zero in the far-field expansion

63 $$\begin{aligned} C^\varepsilon (X,0) \sim {\left\{ \begin{array}{ll} a_- - \dfrac{A^\varepsilon \mathrm{{e}}^{\varepsilon X}}{2\varepsilon (-X)^{3/2}} \quad &{}\hbox {as}~X \rightarrow -\infty ,\\ a_+ - \dfrac{A^\varepsilon \mathrm{{e}}^{-\varepsilon X}}{\varepsilon X^{1/2}} \quad &{}\hbox {as}~X \rightarrow +\infty , \end{array}\right. } \end{aligned}$$

which follows from (59). We shall therefore apply the Wiener–Hopf method to the functions $\overline{F}^\varepsilon _{\pm }(k)$. This is equivalent to applying it to the functions $k\overline{C}_\pm (k)$ due to (61) and (62), since $\overline{F}^\varepsilon _+(k)$ is holomorphic in $\mathfrak {I}(k) > -\varepsilon$ and $\overline{F}^\varepsilon _-(k)$ is holomorphic in $\mathfrak {I}(k) < \varepsilon$, so that these functions are both holomorphic in the overlap strip $-\varepsilon< \mathfrak {I}(k) < \varepsilon$. Before proceeding with the Wiener–Hopf method in the next section, we note that the Abelian Theorem in Appendix 3, together with the identities (61) and (62) (extended to $\mathfrak {I}(k) > -\varepsilon$ and $\mathfrak {I}(k) < \varepsilon$, respectively), gives the far-field behaviour

64 $$\begin{aligned} k\overline{C}^{\varepsilon }_+(k) \sim&iC^{\varepsilon }_O, \quad \overline{F}^\varepsilon _+(k) \rightarrow 0, \quad&\hbox {as}~k\rightarrow \infty ,~\mathfrak {I}(k) > -\varepsilon , \end{aligned}$$

65 $$\begin{aligned} k\overline{C}^{\varepsilon }_-(k) \sim&-iC^{\varepsilon }_O, \quad \overline{F}^\varepsilon _-(k) \rightarrow 0, \quad&\hbox {as}~k\rightarrow \infty ,~\mathfrak {I}(k) < \varepsilon . \end{aligned}$$

#### Wiener–Hopf method

We begin by defining branches of the square roots $(k\pm i\varepsilon )^{1/2}$:

66 $$\begin{aligned} (k+i\varepsilon )^{1/2} =&|k+i\varepsilon |^{1/2}\mathrm{{e}}^{i\arg (k+i\varepsilon )/2},~&\hbox {for}~-\frac{\pi }{2} \le \arg (k+i\varepsilon ) < \frac{3\pi }{2}, \end{aligned}$$

67 $$\begin{aligned} (k-i\varepsilon )^{1/2} =&|k-i\varepsilon |^{1/2}\mathrm{{e}}^{i\arg (k-i\varepsilon )/2},~&\hbox {for}~-\frac{3\pi }{2} \le \arg (k-i\varepsilon ) < \frac{\pi }{2}. \end{aligned}$$

Thus, the square root $(k+i\varepsilon )^{1/2}$ has branch cut $S_- = \{k\in \mathbb {C}:\mathfrak {R}(k) = 0,~\mathfrak {I}(k) \le -\varepsilon \}$, while $(k-i\varepsilon )^{1/2}$ has branch cut $S_+ = \{k\in \mathbb {C}:\mathfrak {R}(k) = 0,~\mathfrak {I}(k) \ge \varepsilon \}$. We then define

68 $$\begin{aligned} (k^2+\varepsilon ^2)^{1/2} = (k+i\varepsilon )^{1/2}(k-i\varepsilon )^{1/2}, \end{aligned}$$

which has positive real part everywhere on the cut plane $\mathbb {C}\setminus (S_+\cup S_-)$.

Now we define the Fourier transform in *X* of $C^{\varepsilon }(X,Y)$ by

69 $$\begin{aligned} \overline{C}^\varepsilon (k,Y) = \int _{-\infty }^\infty C^{\varepsilon }(X,Y)\mathrm{{e}}^{ikX}\,\mathrm {d}X. \end{aligned}$$

Taking a Fourier transform in *X* of (56), we find that

70 $$\begin{aligned} \overline{C}^\varepsilon (k,Y) = B(k)\mathrm{{e}}^{-(k^2 + \varepsilon ^2)^{1/2}Y}, \quad B(k) = \overline{C}^{\varepsilon }_+(k) + \overline{C}^{\varepsilon }_-(k). \end{aligned}$$

We therefore expect $\overline{C}^\varepsilon (k,Y)$ to be holomorphic in the strip $-\varepsilon< \mathfrak {I}(k) < \varepsilon$ except for a simple pole at the origin. The boundary conditions (57) and (58) imply that

71 $$\begin{aligned} -(k^2+\varepsilon ^2)^{1/2}B(k) = \overline{C}^{\varepsilon }_-(k), \end{aligned}$$

so that eliminating *B*(*k*) between (70) and (71), and using (62) and (62) gives the following Wiener–Hopf equation for the functions $\overline{F}^\varepsilon _\pm (k)$:

72 $$\begin{aligned} \left[ 1 + (k^2 + \varepsilon ^2)^{-1/2}\right] \left[ \,\overline{F}^\varepsilon _-(k) - C^{\varepsilon }_O\right] + \left[ \,\overline{F}^\varepsilon _+(k) + C^{\varepsilon }_O\right] = 0 \quad \hbox {for}~-\varepsilon< \mathfrak {I}(k) < \varepsilon . \end{aligned}$$

In order to apply the Wiener–Hopf method to (72), we must find a product factorization of the function $1 + (k^2 + \varepsilon ^2)^{-1/2}$, namely

73 $$\begin{aligned} 1 + (k^2 + \varepsilon ^2)^{-1/2} = \frac{P_+^{\varepsilon }(k)}{P_-^{\varepsilon }(k)}, \end{aligned}$$

where $P_+^\varepsilon (k)$ is holomorphic in some upper half-plane $\mathfrak {I}(k) > \gamma _+$, and $P_-^\varepsilon (k)$ is holomorphic in some lower half-plane $\mathfrak {I}(k) < \gamma _-$, with $-\varepsilon \le \gamma _+ < \gamma _- \le \varepsilon$. The details of this standard factorization are given in Appendix 4 and reveal that suitable $P^\varepsilon _\pm (k)$ may be found with $P^\varepsilon _+(k)$ holomorphic in $\mathbb {C}\setminus S_-$ and $P^\varepsilon _-(k)$ holomorphic in $\mathbb {C}\setminus S_+$. Given the product factorization (73), we may rewrite the Wiener–Hopf equation (72) as

74 $$\begin{aligned} -\frac{\overline{F}^\varepsilon _-(k) - C^{\varepsilon }_O}{P_-^\varepsilon (k)} = \frac{\overline{F}^\varepsilon _+(k) + C^{\varepsilon }_O}{P_+^\varepsilon (k)} \quad \hbox {for}~-\varepsilon< \mathfrak {I}(k) < \varepsilon . \end{aligned}$$

Since both sides of (74) are equal in the overlap strip $-\varepsilon< \mathfrak {I}(k) < \varepsilon$, we deduce from the identity theorem that the right-hand side is the analytic continuation of the left-hand side into the upper half-plane. In the usual way, this allows us to define an entire *G*(*k*), given by

75 $$\begin{aligned} G(k) = {\left\{ \begin{array}{ll} -\dfrac{\overline{F}^\varepsilon _-(k) - C^{\varepsilon }_O}{P_-^\varepsilon (k)} \quad &{}\hbox {for}~\mathfrak {I}(k) < \varepsilon ,\\ \dfrac{\overline{F}^\varepsilon _+(k) + C^{\varepsilon }_O}{P_+^\varepsilon (k)} \quad &{}\hbox {for}~\mathfrak {I}(k) > -\varepsilon . \end{array}\right. } \end{aligned}$$

Using the large-*k* behaviour (64) and (65) of $\overline{F}^\varepsilon _{\pm }(k)$ and the fact that, by construction, $P_{\pm }^{\varepsilon }(k) \rightarrow 1$ as $k\rightarrow \infty$ (see Appendix 4), we deduce that the large-*k* behaviour of *G*(*k*) is given by $G(k) \sim C^{\varepsilon }_O$ as $k\rightarrow \infty$. Then applying Liouville’s theorem — that a bounded, entire function is constant — to *G*(*k*) tells us that $G(k) \equiv C^{\varepsilon }_O$ and we deduce that

76 $$\begin{aligned} \overline{F}^\varepsilon _+(k) = C^{\varepsilon }_O[P^\varepsilon _+(k) - 1], \quad \overline{F}^\varepsilon _-(k) = C^{\varepsilon }_O[1 - P^\varepsilon _-(k)]. \end{aligned}$$

Solving for $\overline{C}^\varepsilon _\pm (k)$ using (61) and (62), and taking the limit $\varepsilon \rightarrow 0^+$, we obtain

77 $$\begin{aligned} \overline{C}_+(k) = \frac{iC_OP_+(k)}{k}~\hbox {for}~\mathfrak {I}(k) > 0, \quad \overline{C}_-(k) = -\frac{iC_OP_-(k)}{k}~\hbox {for}~\mathfrak {I}(k) < 0, \end{aligned}$$

where $$P_\pm (k) := \lim _{\varepsilon \rightarrow 0^+}P_\pm ^\varepsilon (k)$$ and $$C_O = \lim _{\varepsilon \rightarrow 0^+}C^{\varepsilon }_O$$.

We use the behaviour (120) and (121) of $P_\pm (k)$ near the origin that we derive in Appendix 4 to deduce the behaviour of $\overline{C}_\pm (k)$ near the origin which is given by

78 $$\begin{aligned} \overline{C}_+(k) \sim&\dfrac{\mathrm{{e}}^{3\pi i/4}C_O}{k_+^{3/2}} \quad&\hbox {as}~k\rightarrow 0,~\mathfrak {I}(k) > 0, \end{aligned}$$

79 $$\begin{aligned} \overline{C}_-(k) \sim&\dfrac{\mathrm{{e}}^{-i\pi /4}C_O}{k_-^{1/2}} \quad&\hbox {as}~k\rightarrow 0,~\mathfrak {I}(k) < 0, \end{aligned}$$

with $k_+^{3/2}$ and $k_-^{1/2}$ as defined in (54) and (55). We compare (78) and (79) to the asymptotic results (52) and (53) to deduce that the degree of freedom $C_O$ is given by

80 $$\begin{aligned} C_O = \left( \frac{2}{\pi }\right) ^{1/2}. \end{aligned}$$

We note that (80) may also be derived by, for example, inverting $\overline{F}^\varepsilon _+(k)$ to find $F^\varepsilon _+(X)$ for $X > 0$ (cf. Sect. 5.2.4) and then using Laplace’s method to deduce that

81 $$\begin{aligned} F^\varepsilon _+(X) \sim \frac{\pi ^{1/2}C^{\varepsilon }_OP^\varepsilon _-(-i\varepsilon )\mathrm{{e}}^{-\varepsilon X}}{(2\varepsilon X)^{1/2}} \quad \hbox {as}~X \rightarrow \infty . \end{aligned}$$

We may then apply (59) and (60), together with the fact that $P^\varepsilon _-(-i\varepsilon ) \sim (2\varepsilon )^{1/2}$ as $\varepsilon \rightarrow 0^+$, to deduce that

82 $$\begin{aligned} C^{\varepsilon }_O= \frac{A^\varepsilon (2\varepsilon )^{1/2}}{\pi ^{1/2}P^\varepsilon _-(-i\varepsilon )} \sim \left( \frac{2}{\pi }\right) ^{1/2} \quad \hbox {as}~\varepsilon \rightarrow 0^+. \end{aligned}$$

#### Inversion to find the inner mass flux

To find the mass flux in the inner region, we see from (57) that it is sufficient to find *C*(*X*, 0) for $X < 0$. (The full solution *C*(*X*, *Y*) of the leading-order-inner problem is given for completeness in Appendix 5). Since $C(X,0) = C_-(X)$ for $X < 0$, we will invert $\overline{C}^{\varepsilon }_-(k)$ to find $C^\varepsilon _-(X)$ and take the limit $\varepsilon \rightarrow 0^+$. We have83The inversion contour $\Gamma$ lies below the singularities of $\overline{C}^{\varepsilon }_-(k)$ (namely, the branch cut $S_+$ and the pole at $k=0$), so that for $X > 0$ we may close $\Gamma$ in the lower half-plane, where $\mathfrak {R}(-ikX) < 0$, and use Cauchy’s Theorem to obtain

84 $$\begin{aligned} C^\varepsilon _-(X) = 0 \quad \hbox {for}~X > 0. \end{aligned}$$

For $X < 0$, we deform $\Gamma$ into the upper half-plane, where $\mathfrak {R}(-ikX) < 0$, with a ‘keyhole’ incision around $S_+$, writing $P_-^\varepsilon (k) = P_+^\varepsilon (k)/[1+(k^2+\varepsilon ^2)^{-1/2}]$ since $P_+$ is continuous across $S_+$. We note that this encloses the pole at $k = 0$. We obtain thereby, for $X < 0$,

85 $$\begin{aligned} C^\varepsilon _-(X) = \frac{C^{\varepsilon }_OP_+^\varepsilon (0)}{1 + 1/\varepsilon } + \frac{C^{\varepsilon }_O}{\pi }\int _\varepsilon ^\infty \frac{P_+^\varepsilon (it)(t^2-\varepsilon ^2)^{1/2}\mathrm{{e}}^{tX}}{t(t^2-\varepsilon ^2+1)}\,\mathrm {d}t. \end{aligned}$$

We take the limit $\varepsilon \rightarrow 0^+$ and use the expression (119) for $P_+(it)$ derived in Appendix 4, as well as the fact that $P_+^\varepsilon (0) \sim \varepsilon ^{1/2}$ as $\varepsilon \rightarrow 0^+$, to deduce that

86 $$\begin{aligned} C(X,0) = \frac{2^{1/2}}{\pi ^{3/2}}\int _0^\infty \frac{I(t)\mathrm{{e}}^{tX}}{t^{1/2}(1+t^2)}\,\mathrm {d}t \quad \hbox {for}~X < 0, \end{aligned}$$

where the function *I*(*t*) is given by

87 $$\begin{aligned} I(t) = (1+t^2)^{1/4}\exp \left[ -\frac{1}{\pi }\int _0^t \frac{\log (s)\,\mathrm {d}s}{1+s^2}\right] \quad \hbox {for}~t > 0. \end{aligned}$$

Thus, as $\mathrm {Pe_k}\rightarrow \infty$ with $X = \mathrm {Pe_k}(r-1)=\mathrm {O}(1)$, $X < 0$, the inner mass flux is given by

88 $$\begin{aligned} E(1+\mathrm {Pe_k}^{-1}X) \sim \mathrm {Pe_k}^{1/2}C(X,0). \end{aligned}$$

### Conclusions from the matched-asymptotic analysis

The evaporation rate (40) is of order-unity size in the outer region of order-unity width away from the contact line, while the evaporation rate (88) is of size $\mathrm {O}(\mathrm {Pe_k}^{1/2})$ in the inner region of width $\mathrm {O}(\mathrm {Pe_k}^{-1})$ at the contact line. We therefore expect from (16) that the dominant contribution to the total flux out of the drop *Q* comes from the outer region, with

89 $$\begin{aligned} Q = 4\int _0^1 \frac{r\,\mathrm {d}r}{(1-r^2)^{1/2}} + \mathrm {O}(\mathrm {Pe_k}^{-1/2}) = 4 + \mathrm {O}(\mathrm {Pe_k}^{-1/2}) \quad \hbox {as}~\mathrm {Pe_k}\rightarrow \infty . \end{aligned}$$

(We shall present numerical evidence in Sect. 5.4 that the error term in (89) is in fact of size $\mathrm {O}(\log (\mathrm {Pe_k})/\mathrm {Pe_k})$ as $\mathrm {Pe_k}\rightarrow \infty$.)

We recall that the degree of freedom $c_\mathrm{{L}}(\mathrm {Pe_k})$ belonging to the finite-$\mathrm {Pe_k}$ mixed-boundary-value problem (10)–(13) is related to the degree of freedom $C_O$ of the leading-order-inner problem (41)–(44) by the expression (46). Using the expression (80) for $C_O$, obtained from our matched-asymptotic analysis, we find that

90 $$\begin{aligned} c_\mathrm{{L}}(\mathrm {Pe_k}) \sim 1 - \left( \frac{2}{\pi \mathrm {Pe_k}}\right) ^{1/2} \quad \hbox {as}~\mathrm {Pe_k}\rightarrow \infty . \end{aligned}$$

This result is in agreement with the conclusion (25) (which we made after performing a local analysis of the lens and kinetics-based models) about the way in which kinetic effects regularize the mass-flux singularity. In particular, this tells us, via (47), that the evaporation rate at the contact line $E(1^-)$ is given by

91 $$\begin{aligned} E(1^-) \sim C_O\mathrm {Pe_k}^{1/2} = \left( \frac{2\mathrm {Pe_k}}{\pi }\right) ^{1/2} \quad \hbox {as}~\mathrm {Pe_k}\rightarrow \infty . \end{aligned}$$

Thus our matched-asymptotic expansion is in agreement with the numerics for the exact solution; in our prediction (38) for the large-$\mathrm {Pe_k}$ behaviour of $E(1^-)$, we have $\alpha = (2/\pi )^{1/2} \approx 0.798$, which is presented as the horizontal dashed line in Fig. 2c.

From the expressions (40) and (88) for the evaporation rate in the outer and inner regions, respectively, we deduce that a leading-order additive composite expansion for the evaporation rate *E*(*r*), uniformly valid for $0 \le r < 1$ as $\mathrm {Pe_k}\rightarrow \infty$, is given by

92 $$\begin{aligned} E(r) \sim \frac{2}{\pi (1-r^2)^{1/2}} + \frac{(2\mathrm {Pe_k})^{1/2}}{\pi ^{3/2}}\int _0^\infty \frac{I(t)\mathrm{{e}}^{-\mathrm {Pe_k}(1-r)t}}{t^{1/2}(1+t^2)}\,\mathrm {d}t - \frac{2^{1/2}}{\pi (1-r)^{1/2}}. \end{aligned}$$

### Validation of asymptotic results

We shall now validate our leading-order asymptotic predictions against the finite-$\mathrm {Pe_k}$ solutions that we obtained in Sect. 4. We shall consider the predictions for the total flux out of the drop *Q*, the evaporation rate at the contact line $E(1^-)$, and the evaporation rate *E*(*r*) as a function of *r*.

**Fig. 3:**
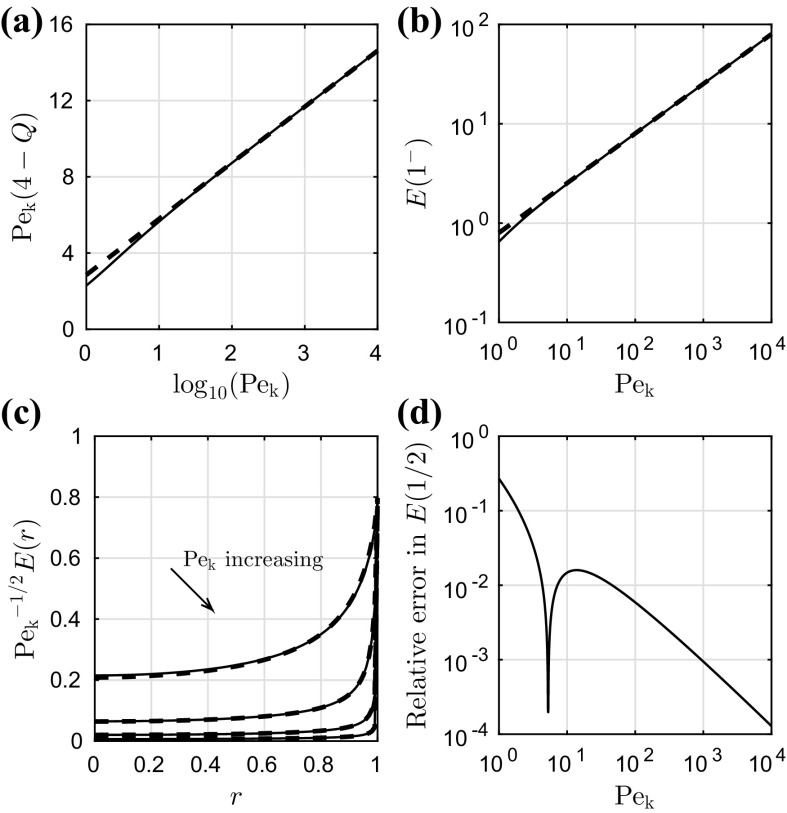
Validation of the leading-order asymptotic results (89), (91), and (92) against the finite-$\mathrm {Pe_k}$ solutions. In **a**, we plot $\mathrm {Pe_k}(4-Q)$ as a function of $\log _{10}(\mathrm {Pe_k})$. The *solid curve* is the finite-$\mathrm {Pe_k}$ solution (36), while the *dashed line* is a linear fit for large values of $\mathrm {Pe_k}$ (between $10^2$ and $10^4$). In **b**, we plot the evaporation rate at the contact line $E(1^-)$ as a function of $\mathrm {Pe_k}$. The *solid curve* is the finite-$\mathrm {Pe_k}$ solution (33), while the *dashed line* is the large-$\mathrm {Pe_k}$ asymptote (91). **c** Scaled evaporation rate $\mathrm {Pe_k}^{-1/2}E(r)$ as a function of *r* for $\mathrm {Pe_k}= 10^1,10^2,10^3,10^4$. The *solid curves* are the finite-$\mathrm {Pe_k}$ solution (32) and the *dashed curves* show the leading-order asymptotic prediction (95). **d** The relative error in *E*(1 / 2) between the finite-$\mathrm {Pe_k}$ solution (32) and the leading-order asymptotic prediction (95).

In Fig. 3a, we take the finite-$\mathrm {Pe_k}$ solution for the total flux *Q* and plot $\mathrm {Pe_k}(4 - Q)$ as a function of $\log _{10}(\mathrm {Pe_k})$. For large values of $\mathrm {Pe_k}$ (between $10^2$ and $10^4$) we fit a linear relationship, which we plot on the same axes. We see that for the physically realistic values of $\mathrm {Pe_k}$ (40 and higher; see Table 1), there is very good agreement between the fit and the data. This gives us confidence that the leading-order asymptotic prediction (89) is correct, but with

93 $$\begin{aligned} Q \sim 4 - \frac{A\log (\mathrm {Pe_k}) + B}{\mathrm {Pe_k}} \quad \hbox {as}~\mathrm {Pe_k}\rightarrow \infty , \end{aligned}$$

where we find numerically that $A \approx 1.28$ and $B\approx 2.85$. We do not investigate further in this paper such higher-order terms.

We plot the finite-$\mathrm {Pe_k}$ solution for the evaporation rate at the contact line $E(1^-)$, given by (33), as a function of $\mathrm {Pe_k}$ in Fig. 3b. On the same axes we plot the leading-order asymptotic prediction (91). We see that there is good agreement between the solutions even for moderately large values of $\mathrm {Pe_k}$. We note that both the form of the asymptote (91) and its validity for moderately large kinetic Péclet numbers are consistent with the observations that we made about the finite-$\mathrm {Pe_k}$ solution following Fig. 2c.

To evaluate numerically the leading-order composite expansion for the evaporation rate (92), we first write the function *I*(*t*) as

94 $$\begin{aligned} I(t) = (1+t^2)^{1/4}\exp \left[ \frac{1}{\pi }\int _t^\infty \frac{\log (s)\,\mathrm {d}s}{1+s^2}\right] , \end{aligned}$$

so that the integrand in (94) is bounded at the endpoints of the integration range. We then make the substitution $t = \tau ^2$ in order to remove the integrable singularity in the integrand of the second term in (92); we obtain, as $\mathrm {Pe_k}\rightarrow \infty$,

95 $$\begin{aligned} \begin{aligned}&E(r) \sim \frac{2^{1/2}[2^{1/2} - (1+r)^{1/2}]}{\pi (1-r^2)^{1/2}} + \frac{2^{3/2}\mathrm {Pe_k}^{1/2}}{\pi ^{3/2}}\int _0^\infty \frac{V(\tau ;r,\mathrm {Pe_k})}{(1+\tau ^4)^{3/4}}\,\mathrm {d}\tau ,\\&\hbox {with}~V(\tau ;r,\mathrm {Pe_k}) = \exp \left[ -\mathrm {Pe_k}(1-r)\tau ^2 + \frac{1}{\pi }\int _{\tau ^2}^\infty \frac{\log (s)\,\mathrm {d}s}{1+s^2}\right] . \end{aligned} \end{aligned}$$

The integrals in (95) are computed in Matlab with the same methods used in the evaluation of (32). We plot the composite evaporation rate (95) as a function of *r* for $\mathrm {Pe_k}= 10^1,10^2,10^3,10^4$ in Fig. 3c. On the same axes we plot the finite-$\mathrm {Pe_k}$ solutions (32); we see good agreement between the two solutions even for only moderately large values of $\mathrm {Pe_k}$. In Fig. 3d, we plot the relative error in *E*(1 / 2) between the finite-$\mathrm {Pe_k}$ solution (32) and the leading-order asymptotic prediction (95). The sharp dip in Fig. 3d is because for $\mathrm {Pe_k}= \mathrm {O}(1)$, the asymptotic prediction is an overestimate, while for large $\mathrm {Pe_k}$, it is an underestimate (i.e. the correction changes sign). For physically realistic values of the kinetic Péclet number (see Table 1), the relative error in *E*(1 / 2) is below 2% and is a decreasing function of $\mathrm {Pe_k}$, illustrating very good agreement between the two solutions (32) and (95).

## Discussion

Our first aim in this paper was to investigate how the mass-flux singularity at the contact line of a thin, evaporating drop is regularized by applying a linear constitutive law on the liquid–gas interface that takes kinetic effects into account. Our second aim was to derive an explicit expression for the evaporation rate.

In Sect. 2, we formulated a model for the transport of liquid vapour within the gas phase, assuming that the vapour concentration is steady, there is no flux of vapour through the solid substrate, the mass flux through the liquid–gas interface is governed by a linear, kinetics-based constitutive law, and the diffusion coefficient and the equilibrium and far-field vapour concentrations are all constant. The model was non-dimensionalized, leaving us with a single dimensionless parameter, the kinetic Péclet number $\mathrm {Pe_k}$ (the ratio of the timescales of diffusive and kinetic effects). We tabulated the values of the physical parameters for hexane, isopropanol, and HFE-7100 and saw that $\mathrm {Pe_k}$ was typically large for all but the smallest drops.

In Sect. 3, we performed a local analysis in the vicinity of the contact line on the kinetics-based model and also on the more standard lens evaporation model (which leads to a mass-flux singularity at the contact line). This demonstrated that the vapour concentration at the contact line $c_\mathrm{{L}}(\mathrm {Pe_k})$ in the kinetics-based model was key to how the mass-flux singularity is regularized, with $c_\mathrm{{L}} \rightarrow 1^-$ as $\mathrm {Pe_k}\rightarrow \infty$, but with $c_\mathrm{{L}} < 1$ for finite $\mathrm {Pe_k}$. This motivated the need to solve the mixed-boundary-value problem formulated in Sect. 2 and determine the degree of freedom $c_\mathrm{{L}}$.

In Sect. 4, we solved the mixed-boundary-value problem and deduced an expression for the mass flux in terms of a set of coefficients that satisfy a system of infinitely many linear algebraic equations. Analysis of the expression for the mass flux confirmed the hypotheses made in Sect. 3 about the degree of freedom $c_\mathrm{{L}}$ and how the mass-flux singularity is regularized by kinetic effects. Our numerical simulations suggested that there was a boundary layer close to the contact line in which the evaporation rate was of size $\mathrm {O}(\mathrm {Pe_k}^{1/2})$ as $\mathrm {Pe_k}\rightarrow \infty$. This motivated us to further analyse the physically relevant limit of large kinetic Péclet number.

In Sect. 5, we performed a matched-asymptotic analysis of our model in the physically relevant regime of large kinetic Péclet number. We found that the asymptotic structure of the problem consists of an outer region away from the contact line, in which the vapour immediately above the liquid–gas interface is at equilibrium to leading order (as is assumed in the lens model). However, there is also an inner region near the contact line, in which kinetic effects enter at leading order. The leading-order-outer problem is equivalent to the lens model, while the leading-order-inner problem was solved readily using the Wiener–Hopf method. We found that the assumption that the vapour immediately above the drop surface is at thermodynamic equilibrium is valid in the outer region, with the mass-flux singularity being regularized in the inner region. We deduced from our leading-order asymptotic solution that $c_\mathrm{{L}} \sim 1 - (2/\pi )^{1/2}\mathrm {Pe_k}^{-1/2}$ as $\mathrm {Pe_k}\rightarrow \infty$, quantifying the way in which kinetic effects regularize the mass-flux singularity. We also constructed a leading-order additive composite expansion and validated this asymptotic prediction by comparison with the solution found in Sect. 4; we found good agreement for physically realistic values of the kinetic Péclet number. Thus, for such values of the kinetic Péclet number, either solution for the mass flux may be used as an input to a model for the evolution of a liquid drop.

The most important direction for future work is to incorporate our expression for the mass flux into a model for the evolution of the liquid drop. This would allow us to obtain predictions for the evaporation time, the evolution of the drop volume (or, equivalently, the dynamic contact angle or drop thickness), and, in the case of a moving contact line, the evolution of the contact-set radius within this model. Previous theoretical work has obtained such predictions for the lens evaporation model (with a mass-flux singularity at the contact line) [11, 12, 41, 42] and for other evaporation models [7, 11, 18, 43–49]. In particular, it would be informative to compare the predictions of this previous work to the corresponding predictions for the model considered here. This comparison would give us some indication of what net result the inclusion of kinetic effects has on the liquid motion beyond regularizing the mass-flux singularity.

For a pinned drop, the evolution of the drop volume is fully described by the global conservation of mass equation (105). We have seen that, in the physically relevant limit when kinetic effects are weak compared to diffusive effects, the leading-order total flux out of the drop per unit time is the same for the kinetics-based model and the lens model.

For a drop with a moving contact line, we expect an important factor in determining the effect of kinetics to be the relative widths of the inner region in which kinetic effects come into play and the region in which the force singularity at a moving contact line is regularized. If the kinetic region is smaller, presumably the only noticeable effect of kinetics is to regularize the mass-flux singularity, while the remainder of the drop dynamics is the same as for the lens model (which we have shown is the leading-order approximation to the kinetics-based model away from the contact line when kinetic effects are weak compared to diffusive effects). On the other hand, if the kinetic region is at least as large as the region in which the force singularity is regularized, we expect that kinetics will have a more significant effect on the drop dynamics. Analysis of the drop dynamics for the lens model [12] suggests that this effect may be through an effective microscopic contact angle (different to both the true microscopic contact angle and the effective one for the lens model) that appears in the contact-line law.

Our analysis assumed that the timescale of vapour diffusion was much shorter than the timescale of interest (set by the liquid evolution). However, there are some situations in which the timescale of diffusion is comparable to the shortest timescale on which mass loss is important [12]. In such cases, Laplace’s equation must be replaced by the unsteady diffusion equation. The resulting problem for the vapour concentration may be solved analytically [50]; we expect the solution on the timescale of vapour diffusion to converge in the long-time limit to the solution of the steady problem. A more thorough study of vapour transport would therefore be an interesting direction for future work. This point is particularly relevant for water, for which it is thought that the effect of the atmosphere may be important [51–53].

We made the assumption that the equilibrium vapour concentration is constant. However, there are many experimentally relevant scenarios in which it is more reasonable to assume that the equilibrium vapour concentration varies with temperature [21, 45, 54] or with the curvature of the interface [20, 24, 26]. In these cases, the appropriate modification of the mass flux is not independent of the drop thickness. A more thorough investigation of these scenarios would be of interest. In Appendix 6, we use the analysis of this paper to determine the range of lengthscales over which it is appropriate to neglect the effect of variations in the equilibrium concentration due to curvature (i.e. the Kelvin effect) compared to kinetic effects.

We also assumed that the problem is axisymmetric. In the non-axisymmetric case, in the large-$\mathrm {Pe_k}$ limit, we expect that the details of the inner region would be the same in each plane perpendicular to the contact line, provided that the contact line is smooth. It would be interesting to investigate this point further and compare the results to previous work on non-axisymmetric drops [48].

The analysis presented in this paper pertains to thin drops, with a small microscopic contact angle $\varPhi$, and is only valid to leading order in the thin-film limit. Since we linearized the boundary condition on the free surface of the drop onto the substrate, the corrections to our analysis are of size $\mathrm {O}(\varPhi )$. While the leading-order prediction is independent of the drop profile, the $\mathrm {O}(\varPhi )$-corrections would depend on the shape of the drop. Dependence on the drop profile is an ingredient in different mass-transfer models, such as those utilizing the Kelvin effect [23, 26].

The expression (2) for the mass flux suggests that the inclusion of kinetic effects also ensures a finite mass flux for thick drops (where the aspect ratio is of order unity). A local analysis of the lens model near the contact line for $0< \varPhi < \pi$ [2, 55], assuming *c* to be continuous at the contact line, implies that, as $r \rightarrow 1^-$,

96 $$\begin{aligned} E(r) \propto (1-r)^{-a}, \quad a = \frac{\pi - 2\varPhi }{2(\pi - \varPhi )}. \end{aligned}$$

Thus, there is a mass-flux singularity at the contact line for $0< \varPhi < \pi /2$. The expression (96) is consistent with the corresponding expression for a thin drop (21) in the limit $\varPhi \rightarrow 0$. On the other hand, a local analysis of the kinetics-based model near the contact line for $0< \varPhi < \pi$ reveals that, as $r \rightarrow 1^-$,

97 $$\begin{aligned} \begin{aligned}&E(r) \sim \mathrm {Pe_k}(1-c_\mathrm{{L}}) \left[ 1 - \frac{\mathrm {Pe_k}}{\sin (\pi -\varPhi )}(1-r)\right] + \ldots \\&\quad -\, \beta (1-r)^{\pi /(\pi -\varPhi )}\cos \left( \frac{\pi ^2}{\pi - \varPhi }\right) , \end{aligned} \end{aligned}$$

where $c_\mathrm{{L}}(\varPhi ,\mathrm {Pe_k})$ and $\beta (\varPhi ,\mathrm {Pe_k})$ are degrees of freedom (the ‘$\ldots$’ indicating that other terms may impinge between those given). Thus the mass flux at the contact line is finite for $0< \varPhi < \pi$. The expression (97) is consistent with the corresponding expression for a thin drop (23) in the limit $\varPhi \rightarrow 0$, provided that

98 $$\begin{aligned} \beta \sim \frac{\mathrm {Pe_k}^2(1-c_\mathrm{{L}})}{\varPhi } \quad \hbox {as}~\varPhi \rightarrow 0. \end{aligned}$$

It would be interesting to investigate more thoroughly how the mass-flux singularity for $0< \varPhi < \pi /2$, $\varPhi = \mathrm {O}(1)$ is regularized by kinetic effects.

## Appendix 1: The liquid phase

In Sect. 1, we noted that the lens model leads to a singularity in the liquid flow at the contact line. In this appendix, we give some brief details about the typical mathematical model for the liquid phase, assuming the flow to be axisymmetric. We use this model to show explicitly that the lens model leads to a singularity in the liquid velocity, while no such singularity is present for the kinetics-based model.

### Formulation

Conservation of mass implies that, in the thin-film limit, the dimensional drop thickness $h^*(r^*,t^*)$ is governed by [10, 12, 56]

99 $$\begin{aligned} \frac{\partial {h^*}}{\partial {t^*}} + \frac{1}{r^*}\frac{\partial {}}{\partial {r^*}}(r^*h^*\overline{u}^*) = -\frac{E^*}{\rho } \quad \hbox {for}~0< r^* < R, \end{aligned}$$

where $t^*$ is time, $\overline{u}^*(r^*,t^*)$ is the depth-averaged radial velocity of the liquid flow, and $\rho$ is the density of the liquid (assumed to be constant). We assume that there are no body forces, the surface-tension $\gamma$ of the liquid–gas interface is constant, and the liquid slips on the substrate according to a Navier slip law [57, 58]. Under these assumptions, an expression for $\overline{u}^*$ is given by [12]

100 $$\begin{aligned} \overline{u}^* = \frac{\gamma }{3\mu }\left( \frac{h^{*2}}{3} + \Lambda h^*\right) \frac{\partial {}}{\partial {r^*}}\left( \frac{\partial ^2h^*}{\partial r^{*2}} + \frac{1}{r^*}\frac{\partial {h^*}}{\partial {r^*}}\right) , \end{aligned}$$

where $\mu$ is the viscosity of the liquid and $\Lambda$ is the slip length (both assumed to be constant). A typical radial lengthscale is given by the initial contact-set radius $R_0$ ($R_0 = R$ if the contact line is pinned). A typical timescale $\tau$ of capillary action may be identified from a balance of the two terms on the left-hand side of the thin-film equation (99) [12]. The thin-film approximation required to derive (99) is valid when the microscopic contact angle $\varPhi \ll 1$ and the reduced Reynolds number $\varPhi ^2\rho R_0^2/\mu \tau \ll 1$.

We non-dimensionalize by setting $r^* = R_0\widehat{r}$, $t^* =\tau t$, $R = R_0s$, $h^* = \varPhi R_0h$, $\overline{u}^* = R_0\overline{u}/\tau$, and $E^* = DM(c_\mathrm{{e}} - c_\infty )\widehat{E}/R_0$ (so that $\widehat{r} = sr$ and $\widehat{E} = E/s$). We obtain thereby the dimensionless thin-film equation given by

101 $$\begin{aligned} \frac{\partial {h}}{\partial {t}} + \frac{1}{\widehat{r}}\frac{\partial {}}{\partial {\widehat{r}}}(\widehat{r}h\overline{u}) = -\alpha \widehat{E} \quad \hbox {for}~0< \widehat{r} < s, \end{aligned}$$

with

102 $$\begin{aligned} \overline{u} = (h^2 + \lambda h)\frac{\partial {}}{\partial {\widehat{r}}}\left( \frac{\partial ^2 h}{\partial {\widehat{r}}^2} + \frac{1}{\widehat{r}}\frac{\partial {h}}{\partial {r}}\right) . \end{aligned}$$

Non-dimensionalization has introduced two dimensionless parameters: the ratio $\alpha$ of the timescales of capillary action and mass loss, and the slip coefficient $\lambda$ that measures the ratio of the drop thickness to the slip length. These dimensionless parameters are given by

103 $$\begin{aligned} \alpha = \frac{3\mu DM(c_\mathrm{{e}} - c_\infty )}{\varPhi ^4\gamma \rho R_0}, \quad \lambda = \frac{3\Lambda }{\varPhi R_{0}}. \end{aligned}$$

Appropriate boundary conditions subject to which to solve the thin-film equation (101) are given by

The two boundary conditions at $\widehat{r} = 0$ (104a, b) are symmetry conditions. The third boundary condition (104c) states that the drop thickness vanishes at the contact line. The fourth boundary condition (104d) states that the dimensionless (small) microscopic contact angle is 1. We note that a local analysis of the thin-film equation (101) and (102) subject to the contact-line boundary conditions (104c, d) implies that there is no flux of liquid through the contact line [12].

We deduce from the thin-film equation (101) and the no-flux boundary conditions that the expression representing global conservation of mass of the drop is given by

105 $$\begin{aligned} \frac{\mathrm {d}{V}}{\mathrm {d}{t}} = -\widehat{Q}; \quad V = 2\pi \int _0^s \widehat{r}h\,\mathrm {d}\widehat{r},~\widehat{Q} = 2\pi \int _0^s \widehat{r}\widehat{E}\,\mathrm {d}\widehat{r}, \end{aligned}$$

where *V* is the (dimensionless) volume of the drop and $\widehat{Q} = sQ$ is the total (dimensionless) mass flux out of the drop per unit time.

### Local analysis

To put our analysis of the lens and kinetics-based models into context, let us first consider the case of no evaporation ($\widehat{E} = 0$). A local analysis of the thin-film equation (101) subject to the boundary conditions (104c, d) reveals that, for a moving contact line,

106 $$\begin{aligned} \overline{u} \sim \dot{s} \quad \hbox {as}~\widehat{r} \rightarrow s^-, \end{aligned}$$

where $$\dot{s} = \mathrm {d}s/\mathrm {d}t$$.

Let us next consider the lens evaporation model. We write the local expansion (21) for the evaporation rate near the contact line in terms of liquid variables:

107 $$\begin{aligned} \widehat{E} \sim \frac{2^{1/2}}{\pi (s-\widehat{r})^{1/2}} \quad \hbox {as}~\widehat{r} \rightarrow s^-. \end{aligned}$$

A local analysis of the thin-film equation (101) subject to the boundary conditions (104c, d) therefore reveals that

108 $$\begin{aligned} \overline{u} \sim \frac{2^{1/2}\alpha }{s^{1/2}(s-\widehat{r})^{1/2}} \quad \hbox {as}~\widehat{r} \rightarrow s^-; \end{aligned}$$

there is an inverse-square-root singularity in the depth-averaged radial velocity at the contact line.

Let us now consider the kinetics-based evaporation model. We write the local expansion (23) for the evaporation rate near the contact line in terms of liquid variables:

109 $$\begin{aligned} \widehat{E} \sim E_\mathrm{{L}} := \frac{\mathrm {Pe_k}[1-c_\mathrm{{L}}(\mathrm {Pe_k})]}{s} \quad \hbox {as} \quad \widehat{r} \rightarrow s^-. \end{aligned}$$

A local analysis of the thin-film Eq. (101) subject to the boundary conditions (104c, d) therefore reveals that

110 $$\begin{aligned} \overline{u} \sim \dot{s} + \alpha E_\mathrm{{L}} \quad \hbox {as} \quad \widehat{r} \rightarrow s^-. \end{aligned}$$

Thus, for the kinetics-based model, there is no singularity in the depth-averaged radial velocity at the contact line.

Using the expression for $\overline{u}$ (102) and the three local expansions (106), (108), and (110), we may make the following deductions. First, the stress singularity at the contact line in the kinetics-based model has the same strength as the one for moving contact lines in the absence of mass transfer (see [59] for further details about the form of the singularity in the latter case). Moreover, this singularity is present for both moving and pinned contact lines (with a different coefficient in each of the two cases). On the other hand, the lens model has a stress singularity at the contact line that is stronger than the classical one for a moving contact line in the absence of evaporation, and this singularity is present even when the contact line is pinned (see [12] for details of the resulting local expansions for *h* at the contact line).

## Appendix 2: Evaporation rate at the contact line

To determine the evaporation rate at the contact line $E(1^-)$, we set $x = \cos (\delta X)$ and $r = \cos (\delta )$ in (32) to obtain

111 $$\begin{aligned} E(\cos (\delta )) = \sum _{n=0}^\infty (2n+1)a_n(\mathrm {Pe_k})I_n(\delta ), \quad I_n(\delta ) = \delta \int _0^1 \frac{\cos [(2n+1)\delta X]}{[\cos ^2(\delta X) - \cos ^2(\delta )]^{1/2}}\,\mathrm {d}X. \end{aligned}$$

We now take the limit $\delta \rightarrow 0^+$ (corresponding to $r\rightarrow 1^-$) of (111). We use the small-argument expansion for the cosine terms in the denominator (but not the numerator) to obtain, as $\delta \rightarrow 0^+$,

112 $$\begin{aligned} I_n(\delta ) = \int _0^1 \frac{\cos [(2n+1)\delta X]}{(1-X^2)^{1/2}} + \mathrm {O}(\delta ^2) = \frac{\pi }{2}J_0[(2n+1)\delta ] + \mathrm {O}(\delta ^2), \end{aligned}$$

where $J_0$ is the Bessel function of first kind of order zero. Truncating the infinite sum in (111) at $n = N(\delta )$, where $N(\delta )$ is an integer chosen such that $1 \ll N(\delta ) \ll \delta ^{-2/3}$ as $\delta \rightarrow 0^+$ (for reasons that will become apparent shortly), we obtain

113 $$\begin{aligned} E(\cos (\delta )) = \frac{\pi }{2}\sum _{n=0}^{N(\delta )} (2n+1)a_n(\mathrm {Pe_k})J_0[(2n+1)\delta ] + {\mathcal {E}}(\delta ) \quad \hbox {as}~\delta \rightarrow 0^+, \end{aligned}$$

where ${\mathcal {E}}(\delta )$ is an error term that we estimate below. Since $\delta N(\delta ) \ll 1$ as $\delta \rightarrow 0^+$ with $N(\delta )$ chosen as described above, we apply the small-argument expansion of the Bessel function, truncated to its first term, and absorb the associated error into ${\mathcal {E}}(\delta )$. We then extend the sum to an infinite number of terms and absorb into ${\mathcal {E}}(\delta )$ the error thus introduced; we obtain

114 $$\begin{aligned} E(\cos (\delta )) = \frac{\pi }{2}\sum _{n=0}^\infty (2n+1)a_n(\mathrm {Pe_k}) + {\mathcal {E}}(\delta ) \quad \hbox {as}~\delta \rightarrow 0^+, \end{aligned}$$

since $J_0(0) = 1$. The first term on the right-hand side of (114) is of order-unity size as $\delta \rightarrow 0^+$. Therefore, provided that the error term ${\mathcal {E}}(\delta ) = \mathrm {o}(1)$ as $\delta \rightarrow 0^+$, the evaporation rate at the contact line $E(1^-)$ is given by (33).

It remains to estimate the error term ${\mathcal {E}}(\delta )$. The first contribution comes from truncating the infinite sum at $n = N(\delta )$. In Sect. 4.2, we present numerical evidence that $a_n = \mathrm {O}(n^{-4})$ as $n\rightarrow \infty$ (see Fig. 2a). Assuming this to be the case, we deduce from the Euler–Maclaurin formula and the boundedness of the Bessel function $J_0$ that the truncation error is no larger than $\mathrm {O}(N^{-2})$ as $N\rightarrow \infty$. Since $N(\delta ) \gg 1$ as $\delta \rightarrow 0^+$, this contribution is of size $\mathrm {o}(1)$, as required. The second contribution comes from using only the leading term in the expansion (112) for $I_n(\delta )$. The error term is of size $\mathrm {O}(\delta ^2)$ as $\delta \rightarrow 0^+$ and there are $N+1$ terms in the sum, so the contribution is of size $\mathrm {O}(\delta ^2N)$. Since $\delta ^2N(\delta ) \ll 1$ as $\delta \rightarrow 0$ with $N(\delta )$ chosen as described above, the second contribution is of size $\mathrm {o}(1)$, as required. The third contribution comes from truncating the small-argument expansion of the Bessel function at its first term. This introduces into the $n^\mathrm{th}$ term in the sum an error of size $\mathrm {O}(n^2\delta ^2)$ as $\delta \rightarrow 0^+$. The contribution from all $(N+1)$ terms is therefore no larger than $\mathrm {O}(N^3\delta ^2)$ as $\delta \rightarrow 0^+$. Since $N(\delta ) \ll \delta ^{-2/3}$ as $\delta \rightarrow 0^+$ (by assumption), the third contribution is of size $\mathrm {o}(1)$, as required. The final contribution comes from extending the sum to an infinite number of terms. This contribution is no larger than $\mathrm {O}(N^{-2})$ as $N\rightarrow \infty$, for the same reasons as for the first contribution, and is therefore of size $\mathrm {o}(1)$ as $\delta \rightarrow 0^+$. We therefore conclude that ${\mathcal {E}}(\delta ) = \mathrm {o}(1)$ as $\delta \rightarrow 0^+$, as required.

## Appendix 3: An Abelian Theorem

We are often interested in the domains of holomorphy of the one-sided Fourier transforms of a function *f*(*x*) (49), as well as their behaviour as $k\rightarrow \infty$. This behaviour is dominated by the behaviour of *f*(*x*) as $x\rightarrow 0$, as described by the following two results [60].

(i) Suppose $f(x) = 0$ for $x < 0$, $|f(x)| < A\mathrm{{e}}^{ax}$ as $x\rightarrow \infty$, for some constants *A* and *a*, *f* is infinitely differentiable for $x > 0$, and $f(x) \sim x^\lambda$ as $x\rightarrow 0^+$, for some $\lambda > -1$. Then $\overline{f}_+(k)$ is holomorphic in $\mathfrak {I}(k) > a$, with

115 $$\begin{aligned} \overline{f}_+(k) \sim \int _0^\infty x^\lambda \mathrm{{e}}^{ikx}\,\mathrm {d}x = \frac{\lambda !}{(-ik)^{\lambda + 1}} \quad \text {as}~k\rightarrow \infty ~\text {in}~\mathfrak {I}(k) > a. \end{aligned}$$

(ii) Suppose $f(x) = 0$ for $x > 0$, $|f(x)| < B\mathrm{{e}}^{bx}$ as $x\rightarrow -\infty$, for some constants *B* and *b*, *f* is infinitely differentiable for $x < 0$, and $f(x) \sim (-x)^\mu$ as $x\rightarrow 0^-$, for some $\mu > -1$. Then $\overline{f}_-(k)$ is holomorphic in $\mathfrak {I}(k) < b$, with

116 $$\begin{aligned} \overline{f}_-(k) \sim \int _{-\infty }^0 (-x)^\mu \mathrm{{e}}^{ikx}\,\mathrm {d}x = \frac{\mu !}{(ik)^{\mu + 1}} \quad \text {as}~k\rightarrow \infty ~\text {in}~\mathfrak {I}(k) < b. \end{aligned}$$

## Appendix 4: Wiener–Hopf product factorization

We now outline the details of the Wiener–Hopf product factorization (73). With our choice of branch for $(k^2+\varepsilon ^2)^{1/2}$, defined by (66)–(68), the left-hand side of (73) is holomorphic and non-zero in the strip $-\varepsilon< \mathfrak {I}(k) < \varepsilon$, and tends to unity as $k\rightarrow \infty$ in this strip. Moreover, the image of the strip under the principal branch of the logarithm, which we denote by $\log$, does not encircle the branch point at the origin. We therefore apply the general method of Carrier et al. [39] and take logarithms to change the problem from a product decomposition to an additive decomposition. We find that

117 $$\begin{aligned} \log P_\pm ^\varepsilon (k) = \frac{1}{2\pi i}\int _{\Gamma _\pm } \frac{\log \left[ 1+(\zeta ^2+\varepsilon ^2)^{-1/2}\right] }{\zeta - k}\,\mathrm {d}\zeta \quad \hbox {for}~\gamma _+< \mathfrak {I}(k) < \gamma _-, \end{aligned}$$

where $\Gamma _{\pm } = \{\zeta \in \mathbb {C}~:~\zeta = x + i\gamma _\pm ,~x\in \mathbb {R}\}$, with the real numbers $\gamma _\pm$ chosen such that $-\varepsilon< \gamma _+< \gamma _- < \varepsilon$. We note that, by construction, $P_\pm ^\varepsilon (k) \rightarrow 1$ as $k\rightarrow \infty$. Following [34], we deform $\Gamma _+$ into the lower half-plane with a ‘keyhole’ incision around the branch cut $S_-$ and $\Gamma _-$ into the upper half-plane with an incision around the branch cut $S_+$. We deduce thereby that $P^\varepsilon _\pm (k)$ is holomorphic for $k\in \mathbb {C}\setminus S_\mp$, with

118 $$\begin{aligned} P_\pm ^\varepsilon (k) = \exp \left( \pm \frac{1}{2\pi }\int _\varepsilon ^\infty \frac{1}{i\sigma \pm k}\log \left[ \frac{1+i(\sigma ^2-\varepsilon ^2)^{-1/2}}{1-i(\sigma ^2-\varepsilon ^2)^{-1/2}}\right] \,\mathrm {d}\sigma \right) \quad \hbox {for}~k\in \mathbb {C}\setminus S_\mp . \end{aligned}$$

It follows from (118) that

119 $$\begin{aligned} P_\pm (\pm it) = t^{\mp 1/2}(1+t^2)^{\pm 1/4}\exp \left( \mp \frac{1}{\pi }\int _0^t \frac{\log (s)}{1+s^2}\,\mathrm {d}s\right) \quad \hbox {for}~t > 0. \end{aligned}$$

By analytic continuation off the imaginary axis, we deduce that

120 $$\begin{aligned} P_+(k) \sim&\mathrm{{e}}^{i\pi /4}k_+^{-1/2} \quad&\hbox {as}~k\rightarrow 0,~\mathfrak {I}(k) > 0, \end{aligned}$$

121 $$\begin{aligned} P_-(k) \sim&\mathrm{{e}}^{i\pi /4}k_-^{1/2} \quad&\hbox {as}~k\rightarrow 0,~\mathfrak {I}(k) < 0, \end{aligned}$$

where $k_+^{-1/2} = k_+^{3/2}/k^2$, with $k_+^{3/2}$ as defined in (54), and $k_-^{1/2}$ is defined in (55).

## Appendix 5: Inversion to find *C*(*X*, *Y*)

In this appendix, we shall invert the Fourier transform in *X* of $\partial C^{\varepsilon }/\partial X (X,Y)$, which by (76) is given by

122 $$\begin{aligned} \overline{F}^\varepsilon (k,Y) = C^{\varepsilon }_O[P^\varepsilon _+(k) - P^\varepsilon _-(k)]\mathrm{{e}}^{-(k^2+\varepsilon ^2)^{1/2}Y}, \end{aligned}$$

Applying the inversion theorem gives

123

where the inversion contour $\Gamma$ runs along the real axis.

For $X < 0$, we deform $\Gamma$ into the upper half-plane, where $\mathfrak {R}[-(k^2+\varepsilon ^2)^{1/2}Y-ikX] < 0$, around the branch cut $S_+$; we also substitute $P^\varepsilon _+(k) - P^\varepsilon _-(k) = P^\varepsilon _+(k)/[1+(k^2+\varepsilon ^2)^{1/2}]$, since $P^\varepsilon _+(k)$ is continuous across $S_+$. After taking the limit $\varepsilon \rightarrow 0^+$, we obtain

124 $$\begin{aligned} \frac{\partial {C}}{\partial {X}}(X,Y) = \frac{2^{1/2}}{\pi ^{3/2}}\int _0^\infty \frac{I(t)[\sin (tY) + t\cos (tY)]\mathrm{{e}}^{tX}}{t^{1/2}(1+t^2)}\,\mathrm {d}t \quad \hbox {for}~X < 0, \end{aligned}$$

with *I*(*t*) as defined in (87). We then integrate (124) with respect to *X* and use the far-field condition (44) to deduce that

125 $$\begin{aligned} C(X,Y) = \frac{2^{1/2}}{\pi ^{3/2}}\int _0^\infty \frac{I(t)[\sin (tY) + t\cos (tY)]\mathrm{{e}}^{tX}}{t^{3/2}(1+t^2)}\,\mathrm {d}t \quad \hbox {for}~X < 0. \end{aligned}$$

We note that it is readily shown that (125) is in agreement with (86) when $Y = 0$.

For $X > 0$, we deform $\Gamma$ into the lower half-plane, where $\mathfrak {R}[-(k^2+\varepsilon ^2)^{1/2}Y-ikX] < 0$, around the branch cut $S_-$; we now substitute $P^\varepsilon _+(k) - P^\varepsilon _-(k) = P^\varepsilon _-(k)/(k^2+\varepsilon ^2)^{1/2}$, since $P^\varepsilon _-(k)$ is continuous across $S_-$. After taking the limit $\varepsilon \rightarrow 0^+$, we obtain

126 $$\begin{aligned} \frac{\partial {C}}{\partial {X}}(X,Y) = \frac{2^{1/2}}{\pi ^{3/2}}\int _0^\infty \frac{\cos (tY)\mathrm{{e}}^{-tX}}{t^{1/2}I(t)}\,\mathrm {d}t \quad \hbox {for}~X > 0. \end{aligned}$$

We then integrate (126) with respect to *X* from $X = 0$, demanding continuity of *C* for $X = 0$, $Y \ge 0$, using (125), to deduce that

127 $$\begin{aligned} C(X,Y) = C(0^-,Y) + \frac{2^{1/2}}{\pi ^{3/2}}\int _0^\infty \frac{\cos (tY)(1-\mathrm{{e}}^{-tX})}{t^{3/2}I(t)}\,\mathrm {d}t \quad \hbox {for}~X \ge 0, \end{aligned}$$

where $C(0^-,Y)$ is given by (125). Although we have constructed a solution that is continuous across $X = 0, Y \ge 0$, we have been unable to verify that *C*(*X*, *Y*) satisfies Laplace’s equation everywhere in $Y > 0$ by a method other than the constructive one presented above. We note that, given the expression (86) for *C*(*X*, 0) for $X < 0$, the inner problem (41)–(44) becomes a Neumann problem, so that the solution may be obtained using standard Green’s function or Fourier Transform methods [61]; however, the resulting form of the solution will inevitably involve a triple integral and therefore be less amenable to quadrature than the solution presented above. Finally, Laplace’s method may be used to show that *C*(*X*, *Y*) satisfies the far-field condition (44) for $\theta = 0$ and $\theta = \pi$, and it is readily checked that *C*(*X*, 0) is infinitely differentiable on $(-\infty ,0)\cup (0,\infty )$ (as postulated in Sect. 5.2.2) and continuous and non-zero at $X = 0$ (as postulated in Sect. 5.2.1).

## Appendix 6: When can the Kelvin effect be neglected?

In this appendix, using the results of our analysis in Sect. 5 of the physically relevant limit in which the kinetic Péclet number $\mathrm {Pe_k}$ is large, we shall answer the question of when it is reasonable to neglect the Kelvin effect compared to kinetic effects.

In order to incorporate the Kelvin effect into our evaporation model, we would assume that the equilibrium vapour concentration $c_\mathrm{{e}}$, rather than being constant, is given by Kelvin’s equation [20, 24, 26],

128 $$\begin{aligned} c_\mathrm{{e}} = c_\mathrm{{s}} - \frac{\gamma Mc_\mathrm{{s}}}{\rho R_\mathrm{{u}}T_\mathrm{in}}\kappa ^* \quad \hbox {on}~z^* = h^*(r^*,t^*), \end{aligned}$$

where $\gamma$ is the surface tension of the liquid–gas interface and $c_\mathrm{{s}}$ is a reference vapour concentration (we may use the values for $c_\mathrm{{e}}$ given in Table 1 as values for $c_\mathrm{{s}}$). In the thin-film limit, the curvature $\kappa ^*$ of the liquid–gas interface is given by

129 $$\begin{aligned} \kappa ^* = \frac{\partial ^2 h^*}{\partial r^{*2}} + \frac{1}{r^*}\frac{\partial {h^*}}{\partial {r^*}}. \end{aligned}$$

The expression (15) for the evaporation rate *E* is then replaced by

130 $$\begin{aligned} E = \mathrm {Pe_k}\left[ (1-c) - \sigma \left( \frac{\partial ^2 h}{\partial r^2} + \frac{1}{r}\frac{\partial {h}}{\partial {r}}\right) \right] , \quad \hbox {where}~\sigma = \frac{\varPhi \gamma M c_\mathrm{{s}}}{\rho RR_\mathrm{{u}} T_\mathrm{in} (c_\mathrm{{s}} - c_\infty )}. \end{aligned}$$

In the outer region, we deduce from (130) that the Kelvin effect may be neglected at leading order provided that $\sigma \ll 1$. This is true provided that the contact-set radius *R* is much larger than a critical value $R_1$. We report the values of $R_1$ for hexane, isopropanol, and HFE-7100 in Table 2, and we see that the assumption $R \gg R_1$ is essentially always satisfied in practice.

**Table 2:** Values of the critical radii $R_1$ and $R_2$ for hexane, isopropanol, and HFE-7100 at 25 $^{\circ }$C and 1 atm

	Hexane	Isopropanol	HFE-7100
$R_1$ (m)	$9.5\times 10^{-11}$	$6.4\times 10^{-11}$	$1.8\times 10^{-10}$
$R_2$ (m)	0.097	0.40	0.30

Provided that the contact-set radius *R* is much larger than $R_1$, the Kelvin effect may be neglected in the outer region. Provided that *R* is much smaller than $R_2$, the Kelvin effect may be neglected in the inner region

In the inner region, where $(1-r) = \mathrm {O}(\mathrm {Pe_k}^{-1})$, $h = \mathrm {O}(\mathrm {Pe_k}^{-1})$, and $(1 - c) = \mathrm {O}(\mathrm {Pe_k}^{-1/2})$ as $\mathrm {Pe_k}\rightarrow \infty$ (assuming the slope of the drop in the inner region to be of order-unity size), we deduce from (130) that the Kelvin effect may be neglected at leading order provided that $\sigma \ll \mathrm {Pe_k}^{-3/2}$. This is true provided that the contact-set radius *R* is much smaller than a critical value $R_2$. We report the values of $R_2$ for hexane, isopropanol, and HFE-7100 in Table 2, and we see that the assumption $R \ll R_2$ is satisfied for all but very large drops. Thus, for drops with a contact-set radius *R* such that $R_1 \ll R \ll R_2$ (and subject to the caveat on the slope mentioned above), we reach the conclusion that it is reasonable to neglect the Kelvin effect compared to kinetic effects at leading order. We note that this conclusion agrees with that made by [20] for a closely related model.
